# Lipid droplets are intracellular mechanical stressors that impair hepatocyte function

**DOI:** 10.1073/pnas.2216811120

**Published:** 2023-04-10

**Authors:** Abigail E. Loneker, Farid Alisafaei, Aayush Kant, David Li, Paul A. Janmey, Vivek B. Shenoy, Rebecca G. Wells

**Affiliations:** ^a^Department of Bioengineering, University of Pennsylvania, Philadelphia, PA 19104; ^b^Center for Engineering Mechano Biology, University of Pennsylvania, Philadelphia, PA 19104; ^c^Department of Mechanical and Industrial Engineering, New Jersey Institute of Technology, Newark, NJ 07102; ^d^Department of Materials Science and Engineering, University of Pennsylvania, Philadelphia, PA 19104; ^e^Department of Medicine, University of Pennsylvania, Philadelphia, PA 19104; ^f^Department of Physiology, University of Pennsylvania, Philadelphia, PA 19104

**Keywords:** mechanobiology, cytoskeleton, chromatin condensation, HNF4α, nuclear deformation

## Abstract

Matrix stiffening and external mechanical stress have been linked to disease and cancer development in multiple tissues, including the liver, where cirrhosis (which increases stiffness markedly) is the major risk factor for hepatocellular carcinoma. Patients with nonalcoholic fatty liver disease and lipid droplet–filled hepatocytes, however, can develop cancer in noncirrhotic, relatively soft tissue. Here, by treating primary human hepatocytes with the monounsaturated fatty acid oleate, we show that lipid droplets are intracellular mechanical stressors with similar effects to tissue stiffening, including nuclear deformation, chromatin condensation, and impaired hepatocyte function. Mathematical modeling of lipid droplets as inclusions that have only mechanical interactions with other cellular components generated results consistent with our experiments. These data show that lipid droplets are intracellular sources of mechanical stress and suggest that nuclear membrane tension integrates cell responses to combined internal and external stresses.

## Significance

Deformation of the nucleus as a result of *extracellular* sources of stress, including increased substrate stiffness, constricted migration, and compression, has been well documented to lead to increased nuclear rupture, changes in gene expression, and accumulation of DNA damage. Lipid droplet accumulation in hepatocytes provides a unique scenario to investigate potential *intracellular* mechanical stresses and sources of nuclear deformation. Our results show that lipid droplets are significant mechanical elements in the cell: deforming the nucleus in a way that impairs hepatocyte function, disrupting cytoskeletal networks, and preventing stiffness-driven alignment of actin fibers.

Nonalcoholic fatty liver disease (NAFLD), typified by lipid-filled hepatocytes, is the most rapidly increasing liver disease globally (1) and a major precursor of cirrhosis (end-stage liver disease). NAFLD is also an increasing cause of hepatocellular carcinoma (HCC) (2), a primary liver cancer leading to 800,000 deaths worldwide per year (3). Cirrhosis, which is characterized by advanced fibrosis, architectural reorganization of the liver, and, notably, increased liver stiffness (4–7), is the main risk factor for HCC and is associated with over 80% of HCC cases (8). Liver stiffness measurements correlate with occurrence of HCC and patient prognosis and are used clinically in HCC surveillance (5–7), consistent with a growing body of mechanobiology literature showing that tissue stiffness contributes to malignancy; mechanical stress from various sources, including matrix stiffness, can lead directly to the accumulation of double-stranded DNA breaks, depletion of DNA repair factors, and chromosomal aberrations (9–12). This suggests a potential role for abnormal mechanics in the development of HCC.

A significant percentage (20 to 63%) of patients with NAFLD-related HCC, however, have noncirrhotic and relatively soft tissues (13–20). We hypothesized that lipid droplets deform the hepatocyte nucleus and serve as *internal mechanical stressors*, even in a soft environment, mimicking the effects of an externally stiff environment. Normal hepatocytes are stiffness sensitive, spreading and losing hepatocyte-specific functional markers in response to increasing substrate stiffness (21–23). Recent research also shows that cells in stiff, cirrhotic livers have deformed nuclei (24), suggesting a link between nuclear deformation and hepatocyte function. The nucleus acts as a central integration site for mechanical stress in multiple cell types, sensing deformation through increases in nuclear membrane tension and dynamically adapting to the local environment (25, 26), potentially through persistent changes in gene expression (27, 28). Deformation can alter gene expression epigenetically and through alteration of 3D chromatin structure, changing the accessibility and transcription of relevant genes (29). Furthermore, nuclear deformation contributes to DNA damage accumulation through increased replication stress (12) and nuclear rupture (9–11, 30–32). The cytoskeleton plays an important role in transmitting external stresses to the nucleus, and disruption of links between nucleus and cytoskeleton is often sufficient to reverse stiffness-mediated effects (24, 33–35).

We previously showed that lipid droplets disrupt normal mechanosensing in primary human hepatocytes (PHH) (36). Our goal in this study was to define experimentally and with theory the interactions between lipid droplets, the cytoskeleton, and the nucleus and to determine their consequences for hepatocyte function. Previous studies of nuclear deformation investigated *extracellular* sources of stress, including increased substrate stiffness, constricted migration, and compression. Lipid droplet accumulation in NAFLD provides a unique scenario to investigate potential *intracellular* mechanical stresses and sources of nuclear deformation.

## Results

For all experiments, PHH were seeded sparsely on soft (500-Pa storage modulus) and stiff (10-kPa storage modulus) polyacrylamide (PAA) gels coated with collagen, which appear mechanically equivalent to normal and cirrhotic liver stiffnesses (36–38). Individual cells not in contact with other cells were analyzed in order to assess the impact of stiffness and to isolate cell–substrate interactions. To induce lipid droplet accumulation, media were supplemented with 0.5% bovine serum albumin (BSA) and 400 µM oleate, a nontoxic fatty acid that is the most common monounsaturated fatty acid in the human diet and is readily taken up and processed by hepatocytes into lipid droplets. 400 µM is comparable to the serum concentration of lipid in obese patients (39) and is nonlipotoxic (36). Control cells were treated with media supplemented with BSA alone.

### Lipid-Loaded Cells Spread Less and Have Smaller Nuclei.

**Fig. 1.**
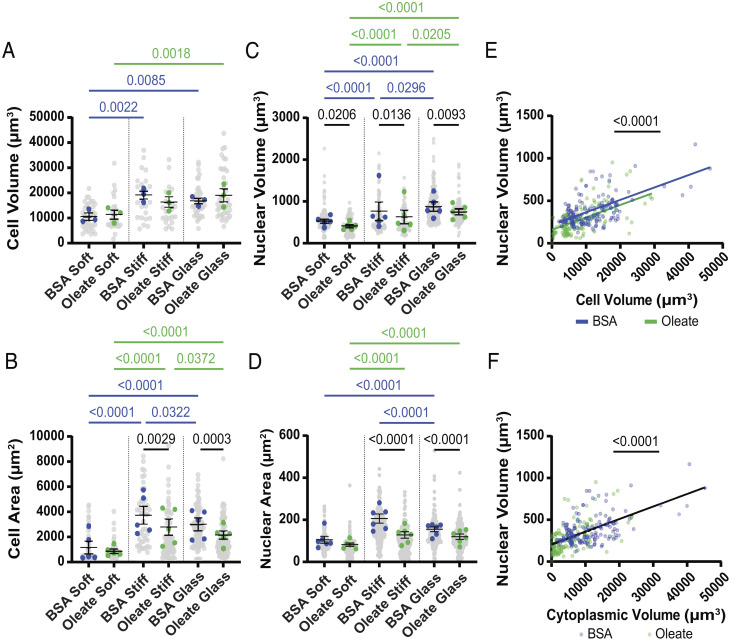
Lipid droplet accumulation decreases nuclear size and disrupts nuclear/cytoplasmic volume ratios. (*A*) Cell volume (*n* = 3), (*B*) cell area (*n* = 5), (*C*) nuclear volume (*n* = 5), and (*D*) nuclear area (*n* = 6) in control and oleate-treated cells on soft PAA, stiff PAA, and glass. (*E*) Scatterplot comparing cell volume and nuclear volume in individual cells, pooled across stiffnesses, fit with linear regression. (*F*) Scatterplot comparing estimated cytoplasmic volume and nuclear volume in individual cells, pooled across stiffnesses, fit with linear regression. Statistics: (*A*–*D*) Data are mean ± SE of *n* independent experiments; *P* values were calculated using two-way ANOVA. (*E* and *F*) Data are values for individual cells from *n* = 3 independent experiments. *P* value was calculated with an F test.

PHH took up a significant amount of oleate when cultured on substrates of all stiffnesses (*SI Appendix*, Fig. S1 *A* and *B*). Although the lipid mean intensity and lipid area fraction were higher in cells on soft compared to stiff substrates (*SI Appendix*, Fig. S1 *A*–*C*), there were no differences in total lipid volume (*SI Appendix*, Fig. S1*D*), suggesting that stiffness does not alter total processing of fatty acids. Hepatocytes increased their volume and spread more on substrates of increasing stiffness, and this relationship was maintained in oleate-treated cells (Fig. 1 *A* and *B*). Compared to controls, oleate treatment reduced cell area on all stiffnesses without altering cell volume, indicating that lipid droplets reduced the ability of cells to spread without increasing overall cell volume. Nuclear cross-sectional area and volume also increased in response to stiffness in all cells; however, lipid-loaded cells had smaller and less spread nuclei than controls on any given stiffness (Fig. 1 *C* and *D*), which was also reflected in a significant downward shift observed in the relationship between cell and nuclear volume in oleate-treated compared to control cells (Fig. 1*E*). This effect is seen for area as well as volume and is thus not explained by lipid-driven changes in cell spreading (*SI Appendix*, Fig. S1*E*); however, given the proportional relationship between nuclear and cell volume (which is linearly related to cytoplasmic volume) for a given cell type (40), it is consistent with a decrease in effective cytoplasmic volume. This was confirmed by plotting nuclear volume against estimated cytoplasmic volume (cell volume–nuclear volume–lipid volume), which showed no difference in the observed relationship between control and oleate-treated cells (Fig. 1*F*). Decreased cytoplasmic volume could potentially impact molecular crowding, osmotic pressures, and reaction rates.

### Lipid Droplets Deform Hepatocyte Nuclei.

**Fig. 2.**
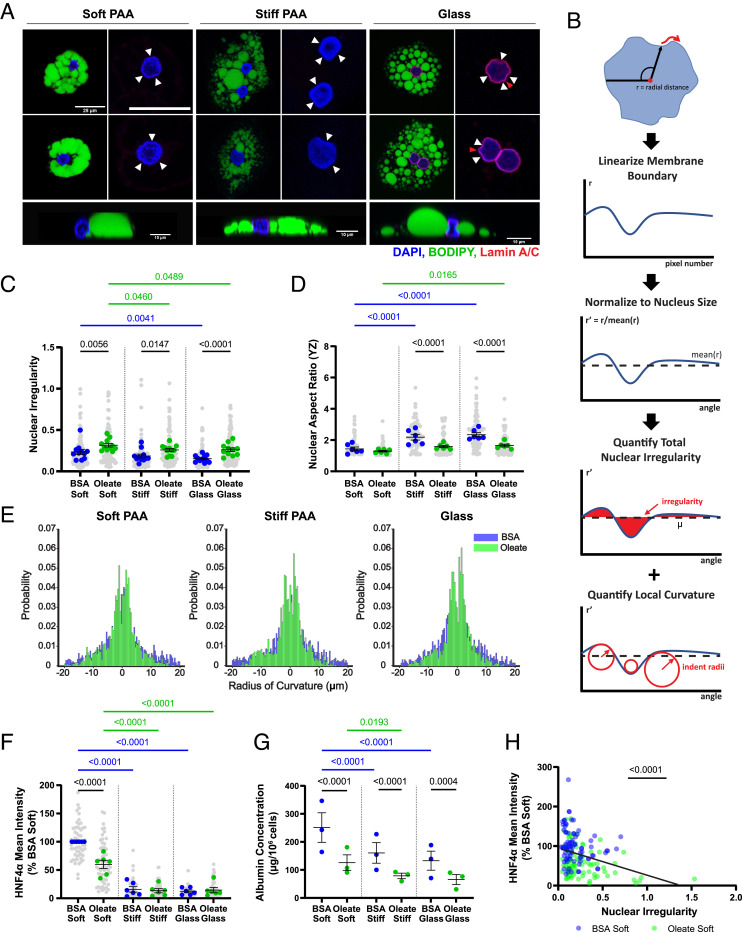
Lipid droplets indent hepatocyte nuclei, impairing hepatocyte-specific functions. (*A*) Nuclei of oleate-treated cells are indented by lipid droplets. Under each heading for stiffness, left column images show representative cells with lipid droplets, and right column images are zoomed-in views of the same cell nucleus with white arrowheads used to emphasize areas of indentation and red arrowheads used to emphasize areas of acute positive curvature. *Bottom* row shows examples of deformation in the YZ plane. Scale is the same for first two rows of images, with the bar = 25 μm. YZ cross-sections have individual scale bars representing 10 μm. DAPI (blue), BODIPY (green), and lamin A/C (red). (*B*) Schematic representation of nuclear irregularity, the parameter used to quantify nuclear deformation in the XY plane. (*C*) Nuclear irregularity (*n* = 5) and (*D*) YZ aspect ratio (*n* = 3) in control and oleate-treated cells. (*E*) Histograms of radii of curvature of nuclear membrane indentations. (*F*) HNF4α mean intensity (*n* = 6, normalized to control on soft) in nuclei of control and oleate-treated cells. (*G*) Concentration of human albumin (*n* = 3) produced by PHH as measured in media of control and oleate-treated cells. (*H*) Scatterplot with linear regression of nuclear irregularity vs. nuclear HNF4α intensity in individual cells on soft substrates. Statistics: (*C*, *D*, *F*, and *G*) Data are mean ± SE of *n* independent experiments. *P* values were calculated using two-way ANOVA with multiple comparisons. (*E*) Data are individual dent radii pooled across cells from n = 3 independent experiments. On each stiffness, control and oleate-treated distributions compared with the K–S test. (*H*) Data are values for individual cells on soft gels from n = 3 independent experiments. *P* value was calculated with an F test.

Lipid droplets deform the nuclear membranes of individual cells on all stiffness substrates (Fig. 2*A*). While classic shape analysis parameters, including circularity, aspect ratio, roundness, and solidity, showed significant differences between control and oleate-treated cells (*SI Appendix*, Fig. S2 *A*–*D*), we developed a deformation parameter that was sensitive to and inclusive of both large- and small-scale deformations to fully capture nuclear membrane shape changes. We wrote a custom MATLAB program that exploits the circularity of hepatocyte nuclei to quantify areas of local deformation. The program identifies the center point of each nucleus and from this point linearizes the nuclear membrane boundary (measuring from the center point to the nucleus boundary), normalizes to the mean radial distance, and plots the normalized radial distance against the angle. From this graph, we calculate the irregularity: the area between the linearized membrane and a line representing the boundary of a perfect circle (Fig. 2*B*). Control cells on all stiffness substrates had a very low irregularity (Fig. 2*C*), but irregularity was increased in oleate-treated cells on all stiffnesses, consistent with the nuclear indentation by lipid droplets we observe (Fig. 2 *A* and *C*). By calculating the local curvature, we also captured the acuteness of indentations in the nuclear membrane (Fig. 2*B*). Histograms of curvature frequency demonstrated that oleate-treated cells on all stiffnesses have a higher frequency of sharp indentations (of both positive and negative curvature) with 1 to 5 micron radii (Fig. 2*E*). The distribution of indentations for control cells is symmetric across the y-axis, consistent with random membrane fluctuations, while the distribution for oleate-treated cells is not, further confirming that these sharp indentations are imposed locally on the nuclear membrane (*SI Appendix*, Fig. S3). Interestingly, there seems to be a higher frequency of high *positive* curvature membrane regions than negative curvature regions in oleate-treated cells, suggesting that high curvature is more likely to occur when the membrane is squeezed between large lipid droplets than as a result of direct indentation by high-curvature lipid droplets (Fig. 2*A*, indented areas indicated by white arrows and sharp positive curvature regions indicated by red arrows and *SI Appendix*, Fig. S3). In contrast to the changes in nuclear irregularity, the nuclear aspect ratio in the YZ plane showed that the nuclei of oleate-treated cells remained similarly rounded on all stiffness substrates and did not exhibit the stiffness-dependent flattening (increased aspect ratio) seen in controls (Fig. 2*D* and *SI Appendix*, Fig. S5*A*). Thus, as the oleate-treated cells became more spread, lipid droplets in the cytoplasm resisted nuclear deformation by cortical actin and stress fibers. This suggests that lipid droplets have dual mechanical roles in the cell: while indenting the nuclei radially, they prevent deformation in the z direction caused by cell spreading.

### Lipid Droplets Impair Hepatocyte Function.

Hepatocyte differentiation is stiffness sensitive, with optimal hepatocyte function occurring when cells are plated on soft substrates (23). Given that stiffness deforms the nuclei of liver cells and that this deformation mediates stiffness-driven phenotypic effects (24), we studied whether the deformation of nuclei by lipid droplets similarly reduced hepatocyte-specific functions. In control and in oleate-treated cells, albumin production and nuclear hepatocyte nuclear factor 4α (HNF4α) intensity decreased significantly with increasing substrate stiffness (Fig. 2 *F* and *G* and *SI Appendix*, Fig. S4*A*), as predicted from the literature, but nuclear HNF4α was also significantly decreased in oleate-treated cells on soft substrates (Fig. 2*F* and *SI Appendix*, Fig S4*A*). HNF4α intensity was negatively correlated with nuclear irregularity on soft substrates, and the relationship was similar for control and lipid-loaded cells (Fig. 2*H*). Lipid-loaded cells also secreted less albumin than controls (Fig. 2*G*). This suggests that on soft substrates, radial deformation of the nucleus by lipid droplets impairs hepatocyte function and shows that the presence of lipid droplets has similar effects as stiff substrates.

**Fig. 3.**
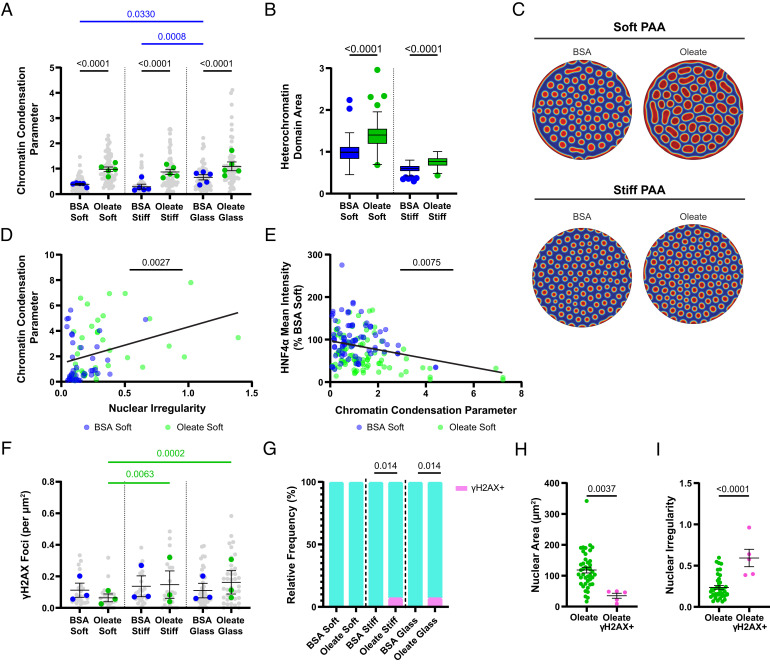
Lipid loading condenses chromatin and can promote DNA damage. (*A*) Chromatin condensation parameter (*n* = 5) of control and oleate-treated cells. (*B*) Quantification and (*C*) visualization of modeled chromatin phase separation in response to mechano-osmotic forces. Circular images represent heterochromatin domain size and organization within simulated cell nuclei for control and oleate-treated cells (red representing compact heterochromatin and blue uncompact euchromatin). The average size of heterochromatin domains over repeated simulations is quantified in *B*. (*D*) Scatterplot with linear regression of chromatin condensation parameter vs. nuclear HNF4α intensity in individual cells on soft substrates. (*E*) Scatterplot with linear regression of nuclear irregularity vs. chromatin condensation parameter in individual cells on soft substrates. (*F*) γH2AX foci (*n* = 3) normalized to nuclear cross-sectional area in control and oleate-treated cells. (*G*) Percentage of cells staining fully positive for γH2AX. (*H*) Nuclear irregularity and (*I*) cross-sectional area of oleate-treated cells depending on γH2AX staining. Statistics: (*A* and *F*) Data are mean ± SE of *n* independent experiments. *P* values were calculated using two-way ANOVA with multiple comparisons. (*D* and *E*) Data are values for individual cells from *n* = 3 independent experiments. *P* value was calculated with an F test. (*G*) Data are percentage of total cells from *n* = 3 independent experiments. *P* values were calculated with the chi-squared test. (*H* and *I*) Data points represent individual cells from the two experiments where fully positive γH2AX nuclei were observed. This subset of cells was compared to the others with an unpaired *t* test.

HNF4α is a key transcriptional regulator in hepatocytes that preferentially associates with high-mobility, decondensed chromatin (41), such that increases in chromatin condensation could decrease expression levels of HNF4α and downstream genes. Therefore, we used image analysis of DAPI-stained nuclei (42, 43) to determine the chromatin condensation state of oleate-treated cells, observing that chromatin condensation increases with both substrate stiffness and lipid loading (Fig. 3*A* and *SI Appendix*, Fig. S4*B*). Chromatin distribution between expressive euchromatin and compacted heterochromatin phases has been proposed to be a result of liquid–liquid phase separation of chromatin (44). However, the role of mechano-osmotic forces in regulating the chromatin reorganizational response of the nucleus is not yet fully understood. To this end, we developed a thermodynamically consistent phase-field model (*SI Appendix*, *Supplemental Methods*) to predict the spatial distribution of chromatin in the nucleus due to either extranuclear mechano-osmotic loads or mechanochemical cues in the form of substrate (or extracellular matrix) stiffness (29).

The model incorporates the following key features: i) energetics of chromatin–chromatin interactions enabling the separation of dissimilar phases, ii) chromatin–lamina interactions enabling formation of lamina-associated domains, iii) diffusion kinetics of nucleoplasm and chromatin enabling a quantity-conserving flow of chromatin, and iv) nonconservative reaction kinetics capturing the methylation or acetylation of chromatin via epigenetic regulation pathways, allowing an interconversion of heterochromatin and euchromatin phases (45). Note that the coarse-grained model is agnostic to molecular origins of the energetic interactions in (i) and (ii), allowing a genome-wide simulation of chromatin organization. Furthermore, while the reactions are assumed to follow first-order kinetics, more complicated kinetics can be incorporated as needed. Consistent with our experimental results showing decreased nuclear volume (Fig. 1*C*), we can simulate the presence of compressive forces on the nuclei due to lipid droplets via the mechano-osmotic compression of the nuclei, driving an outflux of water from the nucleoplasm into the cytoplasm. By allowing a nonzero flux along the periphery of the simulated nucleus, we allow the diffusive exchange of water between the nucleus and the cytoplasm driven by changes in its chemical potential (Eq. 2, *SI Appendix*, Supplemental Methods). The model predicts that oleate-treated cells exhibit increased chromatin compaction on both soft and stiff substrates, increasing the area of compacted heterochromatin domains (Fig. 3 *B* and *C*), consistent with experimental results (Fig. 3*A*).

Chromatin condensation and nuclear irregularity were negatively correlated with HNF4α intensity in oleate-treated cells on soft substrates and positively correlated with each other (Fig. 3 *D* and *E*). This combined with the modeling supports a potential mechanism by which lipid accumulation induces nuclear deformation and decreases nuclear volume, leading to condensed chromatin and reduced expression of HNF4α and downstream hepatocyte-specific genes.

### A Small Subset of Lipid-Loaded Cells Have Increased DNA Damage.

DNA damage can be induced by nuclear deformation in migrating cancer cells (12). We stained with γH2AX to determine the frequency of double-stranded DNA breaks in lipid-loaded cells with nuclear deformation. There was no overall difference in the frequency of double-stranded breaks in control and oleate-treated cells (Fig. 3*F* and *SI Appendix*, Fig. S4*C*). However, a subset of oleate-treated cells on stiff gels and glass had a very high γH2AX-positive signal (Fig. 3*G* and *SI Appendix*, Fig. S4*D*). Interestingly, these nuclei were more irregular and had a smaller cross-sectional area than other oleate-treated cells (Fig. 3 *H* and *I*), suggesting that compression and indentation by droplets increase the likelihood of DNA damage but only in extreme cases.

### Lipid Droplets Disrupt Cytoskeletal Networks.

A previously published chemomechanical model of cytoskeletal nuclear mechanosensing proposes that actin and microtubules act in opposition to determine the flattening and deformation of the nucleus and to generate changes in mechanosensitive signaling (45). We therefore evaluated actin and microtubule networks in lipid-laden hepatocytes.

**Fig. 4.**
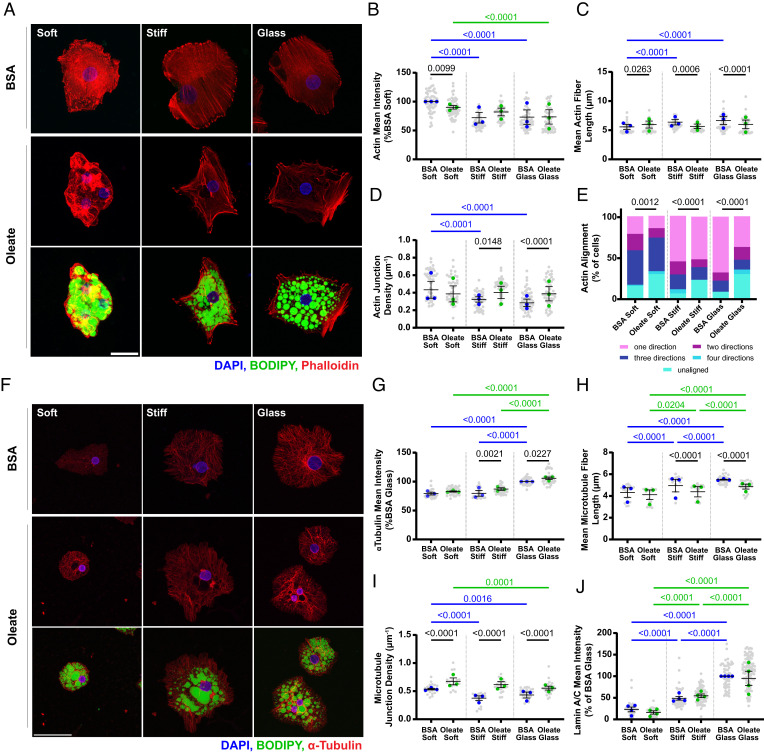
Lipid droplets disrupt cytoskeletal fibers. (*A*) Representative images of actin organization (phalloidin) in control and oleate-treated cells on soft PAA, stiff PAA, and glass. Oleate-treated cells shown with (*bottom* row) and without (*middle* row) lipid droplets. Images are maximum Z projections. Scale bar is 25 μm and the same for all images. DAPI (blue), BODIPY (green), and phalloidin (red). (*B*) Mean phalloidin intensity (normalized to mean of control on soft) for control and oleate-treated cells. (*C*) Mean fiber length and (*D*) junction density of actin fibers. (*E*) Frequency of aligned actin fiber directions in control and oleate-treated cells. (*F*) Representative images of microtubule organization (α-tubulin) in control and oleate-treated cells. Oleate-treated cells are shown with (*bottom* row) and without (*middle* row) lipid droplets. Images are maximum Z projections. Scale bar is 50 μm and applies to all images. DAPI (blue), BODIPY (green), and α-tubulin (red). (*G*) Mean α-tubulin intensity (normalized to mean of control on glass) for control and oleate-treated cells. (*H*) Mean fiber length and (*I*) junction density of microtubules. (*J*) Mean lamin A/C staining (normalized to mean of control on glass) for control and oleate-treated cells. Statistics: (*B*–*D* and *G*–*I*). Data are mean ± SE of *n* = 3 independent experiments. *P* values were calculated using two-way ANOVA with multiple comparisons.(*E*) Data are percentage of total cells from *n* = 3 independent experiments. *P* values were calculated with a chi-squared test comparing control to oleate-treated distribution on each stiffness. (*J*) Data are mean ± SE of *n* = 4 independent experiments. *P* values were calculated using two-way ANOVA with multiple comparisons.

Control cells on soft substrates exhibited high-intensity phalloidin staining, showing actin that was disorganized and diffuse and generally localized to the basal cell membrane (Fig. 4 *A* and *B* and *SI Appendix*, Fig. S5 *A* and *B*). With increasing stiffness, phalloidin intensity (indicative of concentration) and integrated density (indicative of total content) decreased in control hepatocytes and actin fibers became distinct, moving to the apical cell membrane (Fig. 4 *A* and *B* and *SI Appendix*, Fig. S5 *A*–*C*). In oleate-treated cells, actin intensity and integrated density were decreased on soft substrates (Fig. 4*B* and *SI Appendix*, Fig. S5*C*), suggesting that stiffness and lipid loading have similar effects on actin in hepatocytes. Actin fibers were displaced by lipid droplets, particularly in areas with large droplets (Fig. 4*A*). Organization was quantified by assessing fiber length and junction density. Control cells demonstrated a stiffness-dependent increase in actin fiber length and decrease in junction density, consistent with the appearance of stress fibers (Fig. 4 *C* and *D*). Oleate-treated cells had consistent fiber length and junction density without stiffness-related changes but were significantly different than control cells: they had longer fibers and lower junction density than control cells on soft substrates and shorter actin fibers and higher junction density on stiff substrates (Fig. 4 *C* and *D*). We used vector analysis to determine the orientation distribution of actin fibers and to classify cells based on their level of actin fiber alignment (see *Materials and Methods* and ref. 46 for details). For all stiffnesses, oleate-treated cells had lower levels of alignment than controls, with more cells having multiple fiber directions (Fig. 4*E*, note increase in unaligned (teal) and decrease in single alignment direction (pink)). Thus, lipid droplets impose a disorganized and branched actin network structure on soft substrates and prevent stiffness-driven fiber alignment.

Lipid droplets also disrupted microtubules. In both control and oleate-treated cells, α-tubulin intensity and integrated density increased with increasing substrate stiffness (Fig. 4 *F* and *G* and *SI Appendix*, Fig. S5*D*). On all stiffnesses and with oleate treatment, microtubules were distributed throughout the cytoplasm (*SI Appendix*, Fig. S5*E*). However, oleate-treated cells had a higher mean α-tubulin intensity than controls, consistent with a higher microtubule density (Fig. 4*G*). 3D image reconstruction shows microtubules appearing to form cages around the nucleus and individual lipid droplets (Movies S1 and S2). This is supported by fiber network analysis, which shows shorter microtubule length and increased branching frequency in lipid-loaded compared to control cells (Fig. 4 *H* and *I*).

Connections between the cytoskeleton and nucleoskeleton are vital to transmitting external forces to the nucleus. Both control and oleate-treated cells exhibited increasing lamin A/C intensity in response to increased substrate stiffness, as expected with increasing nuclear stress (47). Surprisingly, despite significant cytoskeletal disorganization and disruption, lamin A/C intensity and localization to the nuclear membrane were not affected by lipid loading (Fig. 4*J* and *SI Appendix*, Fig. S5*F*). This suggests that the nuclei of oleate-treated cells remain under increased mechanical stress, even as cell spreading and nuclear compression by stress fibers are reduced.

### Lipid Droplets Reduce Traction Forces.

**Fig. 5.**
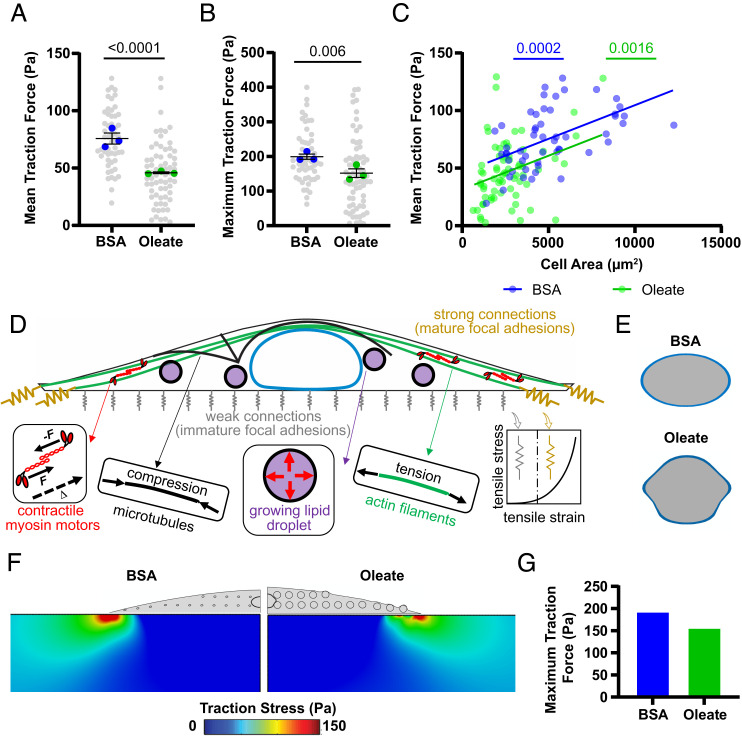
Experimental and modeling approaches show that lipid droplets reduce cell force generation. (*A*) Mean and (*B*) maximum traction forces for control and oleate-treated cells on stiff PAA gels. (*C*) Scatterplot with linear regression of cell area vs. mean traction force in individual cells for control and oleate-treated cells. (*D*) Schematic of chemomechanical model of lipid droplet cytoskeletal interactions in the generation of traction forces. Adapts previously published model (45) and adds lipid droplet growth in the cytoplasm. (*E*) Visualization of lipid droplet–associated nuclear deformation predicted by our mathematical model. (*F*) Visualization and (*G*) quantification of reduced traction forces in lipid-loaded cells as predicted by our mathematical model. Statistics: (*A* and *B*) Data are mean ± SE of *n* = 3 independent experiments. *P* values were calculated using two-way ANOVA with multiple comparisons. (*C*) Data are individual cells from *n* = 3 independent experiments. *P* value was calculated with an F test.

To further investigate the role lipid droplets play in resisting stiffness-driven changes to the cytoskeleton, we measured hepatocyte-generated traction forces (48) and found that lipid loading significantly decreased the mean and maximum traction force (Fig. 5 *A* and *B*). While both control and oleate-treated cells showed a positive correlation between cell area and force generation, the relationship was significantly different for oleate-treated cells (Fig. 5*C* and *SI Appendix*, Fig. S6*A*)—indicating that reductions in traction force are not simply due to a lipid-driven decrease in cell spread area. Furthermore, the force generated in oleate-loaded cells is negatively correlated to lipid density (*SI Appendix*, Fig. S6 *B* and *C*), suggesting that lipid droplets resist cell-generated contractility. These results indicate that lipid accumulation disrupts hepatocyte cytoskeletal networks, rendering them both less able to generate traction forces and less stiffness sensitive than controls.

### Mathematical Modeling Suggests that Mechanical Interaction between Droplets and Other Cellular Components Is Sufficient for Nuclear Indentation and Reduction in Traction Forces.

The previously mentioned chemomechanical model of the cytoskeleton (45) was modified to add lipid droplets to determine whether decreases in traction force could be explained by their physical presence (Fig. 5*D*). The three-dimensional cell model includes the following components: the cytoskeleton, the focal adhesions, and the nucleus (see ref. 45 for details). Lipid droplets were modeled as spherical inclusions that include a thin membrane with internal pressure representing the enclosed fluid. The internal pressure was simulated by applying uniform and outward force spatially perpendicular to the internal surface of the membrane which in turn generates tangential tensile stresses in the membrane representing the surface tension in lipid droplets. The inclusions were arrayed within the cell cytoplasm of circular, well-spread hepatocytes (Fig. 5*D* and *SI Appendix*, Fig S6*D*). Note that the droplets can only have mechanical interactions with other cellular components as described in *SI Appendix*, *Supplemental Methods*. The presence of these growing droplets was sufficient to indent the cell nuclei, further supporting the idea that lipid droplets are a mechanical stress on the nucleus (Fig. 5*E*). For cells with the same area, the addition of lipid droplets reduced the magnitude of traction force that the cells were able to generate, in strong agreement with the experimental results (Fig. 5 *F* and *G*).

#### Altering Contractility Modulates Hepatocyte Function.

**Fig. 6.**
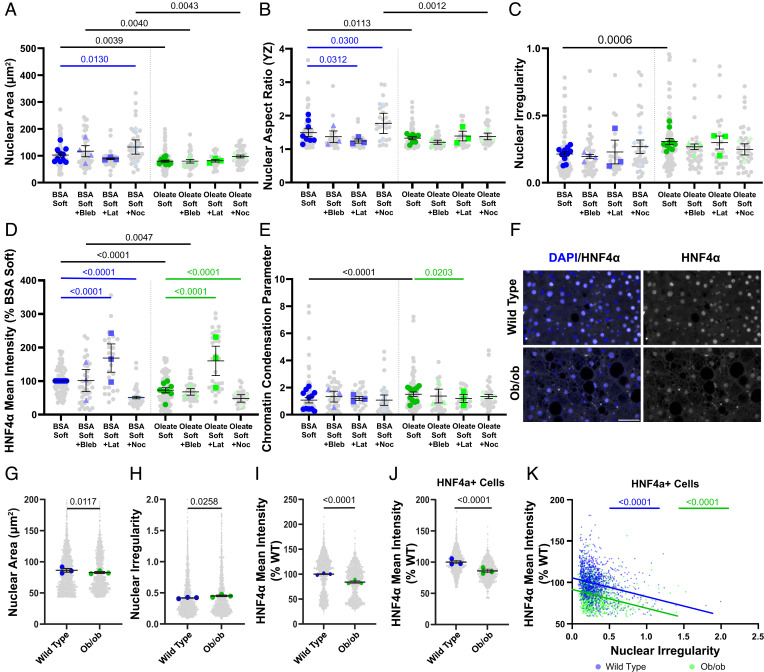
Altering contractility modulates HNF4α expression. (*A*) Nuclear area, (*B*) YZ cross-sectional nuclear aspect ratio, (*C*) nuclear irregularity, (*D*) mean nuclear HNF4α intensity (normalized to control on soft), and (*E*) chromatin condensation parameter in control and oleate-treated cells with or without addition of cytoskeletal drugs on soft PAA. Blebbistatin (Bleb), latrunculin A (Lat), and nocodazole (Noc). (*F*) Representative images of HNF4α staining in wild-type and ob/ob mouse liver tissues. DAPI (blue) and HNF4α (white). Scale bar is 50 μm and is the same for all images. (*G*) Nuclear area, (*H*) nuclear irregularity, and (*I*) mean nuclear HNF4α intensity (normalized to wild type) of all cells in wild-type and ob/ob mouse livers. (*J*) Mean nuclear HNF4α intensity (normalized to wild type) of hepatocytes (gated based on HNF4α intensity) in wild-type and ob/ob mouse livers. (*K*) Scatterplot with linear regression of nuclear irregularity vs. mean HNF4α intensity of hepatocytes from wild-type and ob/ob mouse livers. Statistics: (*A*–*E*) Data are mean ± SE of *n* = 3 for each drug treatment and *n* = 8 for the untreated. *P* values were calculated using two-way ANOVA that applied a main effects model with multiple comparisons. Colored significance values indicate changes due to cytoskeletal drugs, while black significance values are due to lipid loading within a drug treatment group. (*G*–*J*) Data are mean ± SE of *n* = 3 animals. *P* values were calculated with a two-way ANOVA using a main effects model. (*K*) *P* values were calculated with an F test.

We treated lipid-loaded cells with cytoskeletal inhibitors to test whether the deleterious effects of lipid droplets result from active cytoskeletal forces pushing them into the nucleus. Treatment with blebbistatin, which disrupts myosin, and latrunculin A, which disrupts actin, led to decreases in the nuclear area and nuclear aspect ratio in control cells on stiff substrates, as expected given that actin-mediated spreading is inhibited (Fig. 6 *A* and *B* and *SI Appendix*, Fig. S7 *A*–*F*). Nocodazole, which depolymerizes microtubules, led to increases in the nuclear area and aspect ratio, as expected given that microtubule depolymerization has been shown to increase contractility (49) (Fig. 6 *A* and *B* and *SI Appendix*, Fig. S7 *D*–*F*). However, the impact of cytoskeletal disruption was blunted in control cells on soft and oleate-treated cells on all stiffnesses, such that no drug significantly altered the nuclear area or aspect ratio on the PAA gels (Fig. 6 *A* and *B* and *SI Appendix*, Fig. S7 *E* and *F*). Even on glass, where blebbistatin decreased area and nocodazole increased aspect ratio in oleate-treated cells, those changes were significantly less than those in drug-treated control cells (*SI Appendix*, Fig. S7 *E* and *F*). This is consistent with reduced actomyosin contraction in oleate-treated cells making them less sensitive to further pharmacological alteration. In the case of nocodazole, lipid droplets also resist increases in contractility. Surprisingly, nuclear irregularity was not reduced by inhibiting contraction (Fig. 6*C* and *SI Appendix*, Fig. S7*G*), suggesting that contraction is not required for nuclear indentation by lipid droplets. It is also possible that nuclear membrane tension is reduced in drug-treated cells, making it more prone to fluctuation and deformation.

Despite not significantly altering nuclear irregularity, latrunculin treatment increased nuclear HNF4α intensity in both control and oleate-treated cells on soft gels (Fig. 6*D* and *SI Appendix*, Figs. S7*H* and S8*A*). Nocodazole treatment had the opposite effect, leading to decreased HNF4α intensity in both control and oleate-treated cells on soft gels and in control cells on stiff (Fig. 6*D* and *SI Appendix*, Figs. S7*H* and S8*A*). This shows that interactions between lipid droplets and the nuclear envelope are more deleterious in contractile cells. Chromatin condensation analysis in drug-treated cells on soft substrates indicated that latrunculin treatment reduces chromatin condensation in oleate-treated cells but not controls, eliminating the difference seen between control and oleate-treated cells; this suggests that chromatin packing changes may contribute to rescuing HNF4α expression (Fig. 6*E*).

### Lipid Accumulation Deforms Hepatocyte Nuclei and Impairs Function in a Murine Model of Steatosis.

To assess the relevance of lipid droplet–mediated nuclear deformation in an in vivo context, we used a genetic mouse model of NAFLD. The livers of ob/ob mice, which have a mutation in the obese gene (ob) encoding leptin that leads to rapid lipid accumulation in the liver without the development of fibrosis, were stained for HNF4α. Bulk analysis of all cell nuclei found ob/ob mice to have decreased nuclear cross-sectional area, increased nuclear irregularity, and decreased HNF4α intensity compared to lean littermates (Fig. 6 *F*–*I*). Consistent with our in vitro results, nuclear irregularity was negatively correlated with HNF4α expression (*SI Appendix*, Fig. S8*B*). To ensure that this relationship remained true in hepatocytes and was not the result of increased infiltration of immune cells or fibroblasts, we gated cells based on HNF4α intensity to specifically look at likely hepatocytes. Analysis of just these HNF4α+ cells showed that ob/ob mouse hepatocytes had lower HNF4α expression than controls and again expression was negatively correlated with nuclear irregularity (Fig. 6 *J*–*K*). Unlike our in vitro experiments, the regression line was shifted significantly down in ob/ob mice compared to controls, suggesting that there may be other factors associated with lipid accumulation that contribute to reduced HNF4α expression.

## Discussion

We show here that lipid droplets directly indent the hepatocyte nucleus and resist cytoskeletal contraction and that this results in increased chromatin condensation, decreased HNF4α expression, and decreased albumin secretion. Our experimental results were also closely matched by two mathematical models that focused solely on the presence of lipid droplets as incompressible mechanical elements. This shows that lipid droplets are important mechanical elements in the cell, acting as intracellular stressors with effects akin to those of extracellular mechanical stressors like matrix stiffness.

The ability of lipid droplets to deform the nucleus is counterintuitive, given that they are most simply described as an oil-in-water (lipid-in-cytosol) emulsion stabilized by a surfactant (a phospholipid monolayer) (50). The phospholipids, however, create an energy barrier to local surface deformations by increasing the bending energy (50). Additionally, lipid droplet stiffness is a product of the surface tension, which cannot relax without volume changes, while the nucleus is viscoelastic on long timescales. Thus, even though the nucleus is the stiffest organelle in the cell (51), the energy cost of nuclear deformation is less than that of lipid droplet deformation. The magnitude of this barrier can be altered by the phospholipid composition: longer acyl chains and higher membrane concentrations result in a higher energy barrier to deformation. Additionally, different membrane phospholipids have an inherent curvature, and enrichment with negatively curved lipids, such as cholesterol, can promote the fusion of small droplets into larger droplets, which we have seen to be more deleterious (36). This is a potential mechanism for interaction between the biochemical and physical effects of lipid accumulation and suggests that lipid droplet composition may modulate the degree of nuclear disruption. Modeling lipid droplets as growing incompressible spheres recapitulated the nuclear deformation we see in vitro, indicating that they can exert direct force on the nucleus.

The nucleus is an important mechanosensor both in mediating short-term responses to mechanical stimuli and in encoding a long-term “mechanical memory”. Almost all research on the impact of mechanics on the nucleus has used extracellular sources of mechanical stress. Important findings have included the demonstrations that tension exerted on the nuclear membrane can dilate nuclear pores, leading to the translocation of mechanosensitive transcription factors (33, 52); that cells sense deformation through increases in nuclear membrane tension and dynamically adapt in constricted environments (25, 26); and that extreme or persistent levels of nuclear deformation contribute to disease, increasing instances of DNA damage accumulation (9–12, 31). Here, we have shown that cells respond to internal sources of mechanical stress in a similar manner, and we have identified nuclear deformation as a potential integration point for the response to combined internal and external forces. This is especially interesting given the disruption and reorganization of the cytoskeletal network caused by lipid loading. We have shown that lipid-laden hepatocytes are partially resistant to stiffness-driven morphological changes (cell spreading and nuclear flattening), and their nuclei are more resistant to cytoskeletal disruption than control cells. Despite reducing cytoskeletal tension, lipid droplets indent nuclei directly and preserve lamin A/C levels, potentially by maintaining high nuclear membrane tension even as force transmission through the cytoskeleton is disrupted. This is consistent with our previous work showing that large lipid droplets, which induced larger amounts of nuclear deformation, increase nuclear translocation of YAP both in vitro and in human samples (36). These results may also help to explain why fat tissue, which is rheologically soft, has higher lamin expression than tissues of similar stiffness (47) as regulation of lamin A levels evolved to protect nuclei from mechanical stress.

Our results are also consistent with the few published studies of intracellular mechanical stress in different contexts. Research has shown that nuclear membrane fluctuations and chromatin organization can be altered by cell-generated forces (53–55), while work in HeLa cells has shown that large nondeformable cytoplasmic inclusions can greatly increase instances of cell death (56). Importantly, our disease model falls in severity somewhere between these models; lipid accumulation leads to a pathological level of chromatin condensation and a resulting loss of function but does not lead to cell death. Contemporaneous work by colleagues looking at lipid droplets in motile cell lines also supports the role of lipid droplets as internal nuclear stressors and again suggests that cell damage is increased by combined internal and external forces (57).

Our work has important implications for understanding hepatocyte function in NAFLD, particularly the demonstration that nuclear indentation by lipid droplets on soft substrates reduces expression of HNF4α in a manner proportional to the magnitude of deformation. HNF4α is considered the master regulator of hepatocyte function critical for maintaining hepatocyte differentiation and liver health (58, 59). HNF4α expression can be further modulated by altering the contractility of cells with cytoskeletal drugs. On soft substrates, decreasing contractility with latrunculin increased HNF4α in control and oleate-treated cells, fully reversing any reductions due to lipid loading, while increasing contractility with nocodazole did the opposite. Treatment with cytoskeletal drugs also eliminated any correlation between nuclear irregularity and HNF4α expression, suggesting that nuclear deformation is only deleterious when it increases nuclear membrane tension. We have also shown that the relationship between lipid accumulation, nuclear deformation, and HNF4α expression was maintained in tissue. Ob/ob mice are resistant to fibrosis and do not develop more advanced NAFLD without additional injury, making them a good model for studying the earliest stages of simple steatosis; our results suggest even this “bland” type of lipid accumulation can impair liver function due to nuclear stress. Other diet-based models of progressive NAFLD have shown that HNF4α is a central regulator of the progression of steatosis to nonalcoholic steatohepatitis (NASH) (60, 61) and in the development of NAFLD-related HCC (62). In human patients, progressive loss of HNF4α has recently been shown to be a crucial step in the progression of chronic liver diseases and HCC (63). Our research suggests that the lipid-associated nuclear deformation seen in human NAFLD biopsies (64) could be a contributing factor to this loss.

Importantly, we saw an increase in double-stranded DNA breaks (as indicated by γH2AX) in only a few lipid-loaded cells, suggesting that short-term deformation by lipid droplets is a less severe mechanical stress on the nucleus than external confinement. Lipid accumulation alone may not be sufficient to increase DNA damage but may sensitize nuclei to damage from external mechanical stresses such as increased tissue stiffness. This is consistent with the clinical progression of NAFLD: while NAFLD patients are at a significantly increased *relative* risk of HCC, their absolute risk remains low, lower than would be expected if the cells were regularly incurring significant DNA damage. In our experiments, hepatocytes were only loaded with lipid for 48 h, while an intact fatty liver would see lipid accumulation and persistence over many years, such that even a small increased risk of damage could lead to cancer over a long enough time.

More broadly, this research suggests that stiff intracellular inclusions could be sources of mechanical stress in disease. Glycogen and lysosomal storage diseases affect multiple organ systems including the liver, skeletal muscle, heart, and brain and lead to the accumulation of large cytoplasmic inclusions (65, 66). It is therefore possible that nuclear deformation from intracellular stresses may contribute to disease progression in these cases as well. Overall, our results and others highlight the importance of looking at changing mechanical stresses in disease, at both the cell and tissue levels, as they are often characterized by multiple and competing disruptions. How cells respond to combinations of forces, both internal and external, is currently not well understood and is an area ripe for additional research.

## Materials and Methods

### PAA Gel Preparation.

PAA gels with a storage modulus of 500 Pa or 10 kPa were made as described (67). Cells were also cultured on glass (~GPa) as a nonphysiologically stiff control. Glass coverslips and PAA gels activated with sulfo-SANPAH (Thermo Fisher Scientific, Waltham, MA) were incubated with 0.1 mg/mL rat tail collagen type 1 (Corning, Edison, NJ) for 2 h at room temperature to coat the surface with extracellular matrix ligands and enable cell adhesion. Gels were sterilized by UV exposure for 30 min.

### Cell Culture.

Cryopreserved PHH from single donors (BD Gentest, Tewksbury, MA; Lonza, Walkersville, MD; and Thermo Fisher Scientific) were used for all in vitro experiments. PHH were thawed according to the supplier’s instructions using Cryopreserved Hepatocyte Recovery Medium (CM7000; Thermo Fisher Scientific), resuspended in Williams’ Medium E (MilliporeSigma, Burlington, MA) with plating supplements (CM3000; Thermo Fisher Scientific), and plated at a density of 50,000 cells/cm^2^. One day after seeding, PHH were serum starved overnight in serum-free Williams’ Medium E with maintenance supplements (CM4000; Thermo Fisher Scientific). Cells were then incubated for 48 h in DMEM supplemented with 0.5% BSA (free fatty acid free; MilliporeSigma) and 1% penicillin–streptomycin with or without the addition of 400 μM sodium oleate (68, 69).

For albumin measurements, media were collected at the end of 48 h of lipid loading, and albumin concentration was measured with a commercially available human albumin ELISA kit (Invitrogen, Waltham, MA).

After 48-h lipid loading, media were changed to DMEM with 1% penicillin–streptomycin with or without the addition of cytoskeletal drugs: 5 µM blebbistatin (MilliporeSigma), 5 µM latrunculin (MilliporeSigma), or 10 µM nocodazole (Cayman Chemicals, Ann Arbor, MI). Cells were treated for 4 h before fixation.

### Animal Studies.

Livers were harvested from ob/ob mice and wild-type littermates at 12 w, fixed in 10% neutral buffered formalin (Fisher Scientific, Hampton, NH), and paraffin-embedded. Sections were stained with anti-HNF4α antibody tagged with Alexa Fluor 555 (1:100, ab217518; Abcam, Cambridge, UK) and imaged at 40× using a Zeiss Axio Observer 7.

### Immunofluorescence Staining and Microscopy.

Following culture, cells were fixed with 4% paraformaldehyde (Thermo Fisher Scientific) for 15 min and permeabilized with 0.1% Triton X-100 in phosphate buffered saline (PBS) for 15 min. For lamin A/C and HNF4α staining, the permeabilization step was increased to 0.5% Triton X-100 in PBS. Samples were blocked with 5% normal goat serum (Jackson ImmunoResearch Laboratories, West Grove, PA) in PBS for 1 h. Primary antibody for α-tubulin (1:1,000, T6199 MilliporeSigma), lamin A/C (1:200, sc-7292, Santa Cruz), or γH2AX (1:100, 05-636-25UG, MilliporeSigma) was applied for 2 h at room temperature (RT) followed by secondary antibody (fluorophore-conjugated IgG, 1:500; Jackson ImmunoResearch Laboratories) for 1 h at RT. Preconjugated Alexa Fluor 647 HNF4α (1:100, ab217073, Abcam) was applied for 2 h at RT.

To stain neutral lipids, cells were incubated with BODIPY (1:1,000; Thermo Fisher Scientific) for 1 h at 37 °C. Actin was stained with phalloidin (1:50; Invitrogen) for 30 min at RT and nuclei with DAPI (1:5,000; Thermo Fisher Scientific) for 15 min at RT. Samples were washed with PBS between steps. Samples were mounted with aqueous mounting medium (KPL, Gaithersburg, MD). Images were taken using a Leica TCS SP8 laser scanning confocal microscope with a 40× water immersion lens.

### Image Analysis.

Fluorescence intensity of various proteins was analyzed with FIJI. Fluorescence intensity and integrated density were quantified within individual cells manually segmented by tracing cell outlines. Nuclei were segmented by smoothing and thresholding the DAPI-stained regions and then filling the holes of the binary image. Segmented nuclei were postprocessed in MATLAB to quantify the nuclear irregularity or used as ROIs within FIJI to measure the nuclear area and immunofluorescent intensity. γH2AX foci within the nucleus were counted manually in FIJI. Cell volumes were calculated using the 3D Imaging Toolbox for FIJI or MATLAB on confocal Z stacks of phalloidin staining.

Nuclear irregularity and local membrane curvature were quantified with a custom MATLAB program (available at https://github.com/aloneker/NuclearIrregularity). The nuclear YZ aspect ratio was calculated by reslicing confocal Z stacks, taking the maximum projection and thresholding to segment each nucleus. Chromatin condensation analysis was performed according to a published method (42, 43).

Actin and microtubule fiber length and branching density were determined using the Ridge Detection plugin for FIJI. The junction density was calculated by dividing the total number of junctions in the cell by the total fiber length. Actin fiber alignment was further analyzed in MATLAB using a previously published method: the FINE alignment analysis (46).

More details are included in *SI Appendix*.

### Statistics and Data Analysis.

All data represent experiments using hepatocytes from at least three different biological donors. In each plot, measurements from individual cells are shown in gray, while the average measurements from the biological replicates are shown with the colored dots. To avoid overconfidence, statistics were performed on the biological replicates wherever possible. Details on specific tests are included in figure legends and *SI Appendix*, *Supplemental Methods*.

## Supplementary Material

Appendix 01 (PDF)

Movie S1. Z-stack of microtubule organization in control primary human hepatocyte on glass. **Nuclei stained with DAPI (Blue), microtubules with α-tubulin (Red) and lipid with BODIPY (Green). Scale bar is 15μm.**

Movie S2. Z-stack of microtubule organization in oleate-treated primary human hepatocyte on glass. **Nuclei stained with DAPI (Blue), microtubules with α-tubulin (Red) and lipid with BODIPY (Green). Scale bar is 15μm.**

## Data Availability

All study data are included in the article and/or *SI Appendix*.
