# Supplementary material for: Lipid droplets are intracellular mechanical stressors that impair hepatocyte function
Source: Proc Natl Acad Sci U S A. 2023 Apr 10;120(16):e2216811120. doi: 10.1073/pnas.2216811120 (PMC10120019; doi:10.1073/pnas.2216811120)
Supplement: Supplementary file 1 — Appendix 01 (PDF) [file pnas.2216811120.sapp.pdf]

Lipid droplets are intracellular mechanical stressors that impair hepatocyte function

Abigail E. Loneker[1,2], Farid Alisafaei[2,3], Aayush Kant[2,4], David Li[2,6], Paul A. Janmey[2,5], Vivek B. Shenoy[2,4], Rebecca G. Wells[1,2,6]

[1]Department of Bioengineering, University of Pennsylvania; Philadelphia, Pennsylvania, 19104, USA.

[2]Center for Engineering Mechanobiology, University of Pennsylvania; Philadelphia, Pennsylvania, 19104, USA.

[3]Department of Mechanical and Industrial Engineering, New Jersey Institute of Technology; Newark, New Jersey, 07102, USA

[4]Department of Materials Science and Engineering, University of Pennsylvania; Philadelphia, Pennsylvania, USA.

[5]Department of Physiology, University of Pennsylvania; Philadelphia, Pennsylvania, USA.

[6]Department of Medicine, University of Pennsylvania; Philadelphia, Pennsylvania, USA.

*Corresponding author: Rebecca G. Wells
Email: rgwells@pennmedicine.upenn.edu

This PDF file includes:

Supporting Text
Figures S1 to S6
Legends for Movies S1 and S2
SI References

Other supporting materials for this manuscript include the following:

Movies S1 to S2

Supporting Information Text

Supplemental Methods

Cell Culture - Lipid Loading

To solubilize the fatty acids and facilitate uptake by PHHs, sodium oleate was preconjugated to BSA. 20 mM oleic acid solution was prepared in 0.01 M NaOH and incubated for 30 min at 70°C. Next, the solution was diluted to 4 mM in 5% FFA-free BSA in PBS and incubated at 37°C for 10 min. The fatty acid-BSA solution was then mixed 1:9 with serum-free DMEM with 1% penicillin-streptomycin to obtain a 400 μM fatty acid, 0.5% BSA solution in DMEM.

Animal Studies

All animal work was carried out in strict accordance with the recommendations in the Guide for the Care and Use of Laboratory Animals of the National Institutes of Health. Animal protocols were approved by the Institutional Animal Care and Use Committee of the University of Pennsylvania (protocol #804031). Ob/ob mice were obtained from the Jackson Laboratories (strain #000632) and were housed in a temperature-controlled environment with appropriate enrichment, ad libitum feeding of standard rodent chow and water, and 12h light/dark cycles. Euthanasia was carried out by CO_2 inhalation followed by exsanguination.

For immunostaining, slides were subjected to antigen retrieval in 10 mM sodium citrate buffer (pH 6.0) using a pressure cooker, blocked with StartingBlock T20 (PBS) Blocking Buffer (Fisher Scientific, Hampton, NH) for 10 min at room temperature, and incubated overnight at 4°C with rabbit anti-HNF-4-alpha antibody tagged with Alexa Fluor 555 (1:100, ab217518; Abcam, Cambridge, UK), 0.1% bovine serum albumin (Sigma Aldrich, St. Louis, MO), and 0.2% Triton X-100 (Sigma Aldrich, St. Louis, MO) in PBS. Sections were then incubated with 0.5 μg/mL DAPI (Thermo Fisher Scientific, Waltham, MA) in dH_2O for 10 min at room temperature to stain for nuclei.

Immunofluorescence Staining and Microscopy

Images of fixed cells were taken with a Leica TCS SP8 laser scanning confocal microscope with 40X water immersion lens. (numerical aperture: 1.1, XY resolution 177.25nm, Z resolution 377.06nm). Confocal z-stacks for volume measurements and cytoskeletal analysis used a system optimized z-step size of 0.42 μm. An additional 10X digital magnification was used to zoom in on individual nuclei for measurements of irregularity, γH2AX foci, and lamin A/C and HNF4α intensity. To ensure quantitative imaging, samples for all groups in an experiment were stained simultaneously and imaged at the same laser intensity during a single imaging session. Laser intensity was kept similar between each imaging session. Replicates were normalized to a single experimental group (noted in each case in the y-axis label, ex. % BSA Glass) to account for any biological variation and staining variability.

Quantification of Cell and Nuclear Volume

Reconstruction of confocal z-stacks is a common method for estimating volume (1, 2) and we confirmed the validity of confocal volume measurements by imaging 15μm FocalCheck Beads (Thermo Fischer Scientific), where we found this measurement had a less than 1% error in both the green and red laser channels (Fig S1F). The cell boundary was segmented by applying smoothing and thresholding the phalloidin stain in each slice. The binary images were processed to fill holes to generate an ROI inclusive of the entire cytoplasmic volume. Individual cells were then segmented using the Simple Segmentation Tool applied to the thresholded stack and cell volumes were calculated using the 3D ROI Manager. Nuclear volumes were calculated similarly with the DAPI channel used to segment the cell nuclei. Specifically for the cell vs nuclear volume and cytoplasmic vs nuclear volume plots, volumes were analyzed using a MATLAB program that would calculate the cell, nuclear, and lipid volume in each cell. This was done first by smoothing and thresholding the phalloidin channel of 3D confocal image stacks, which was used to generate a label matrix to identify individual cells. 3D image properties (regionprops3) were used to calculate cell volume. The cell label matrix was then used to calculate the nuclear and lipid volume (in the DAPI and BODIPY channels respectively) within each cell ROI, applying a mask of each cell and

then calculating volume with regionprops3. Cytoplasmic volume was estimated by subtracting nuclear and lipid volume from the cell volume.

Measurements of Nuclear Irregularity

Binary images of the cell nuclei were generated in FIJI as described and read into MATLAB. The MATLAB Imaging toolbox was used to identify the nuclear boundary and the center of each nucleus. The program linearized the membrane boundary by measuring the distance between the center point and each point along the boundary and plotted this against the angle (see Figure 2b for schematic). To account for the difference in cell area, the linearized membrane boundary was normalized to the mean radius of the nucleus. The program then calculated the nuclear irregularity: the area between the membrane boundary and a perfect circle with the same mean radius. The membrane boundary is smoothed with a loess filter and points of inflection are estimated using two finite differences (analogous to taking the second derivative of a continuous function). Segments of the membrane boundary between two inflection points were then fit with a circle using the Pratt method (6). The radii of curvature along the membrane was collected for each cell and pooled within a group and the probability distribution was estimated with a histogram.

Quantification of Chromatin Condensation

Chromatin condensation analysis was performed according to a previously published method (3, 4). In brief, the raw images were down-sampled and the intensity redistributed before a Sobel edge detection filter was applied. This was followed by automatic thresholding and morphological thinning as well as removal of the nuclear outline. Finally, the chromatin condensation parameter is the number of remaining edge pixels divided by the nuclear area. Detailed methods, including the exact MATLAB program used, representative images of intermediate steps, and validation can be found in SI references 3 and 4.

Quantification of Cytoskeletal Fiber Analysis

Actin and microtubule fiber length and branching density were determined using the RidgeDetection plugin for FIJI. The maximum z-projection of confocal z-stacks was taken and individual cells segmented. The individual cells were then analyzed for mean fiber length, total fiber length, and total number of junctions with the RidgeDetection plugin, using automated fiber detection and analysis (please see FIJI documentation for additional details). The junction density was calculated by dividing the total number of junctions in the cell by the total fiber length. Actin fiber alignment was further analyzed in MATLAB using a previously published method: the FINE alignment analysis (5). The orientation distribution for each cell was determined with Fourier-based image analysis and then the cumulative orientation distribution was fit with a sum of sigmoid functions. Each sigmoid function represents a “fiber family” of pixels aligned in a specific direction. Cells were then classified based on the number of fiber families detected. This method does not rely on detecting fibers, but instead determines orientation on a pixel-by-pixel basis, avoiding the issues associated with automated fiber segmentation. Additional details of this method, including schematics and validation experiments, can be found in SI reference 5 .

Distribution of actin and microtubule density as a function of height was determined by measuring the integrated density of staining in each slice of the stack and dividing by the total integrated density to determine the normalized distribution from bottom to top of the cell. The normalized distribution was resampled in MATLAB to normalize to the cell height and averaged across all cells, then fit with a smoothing spline to visualize distribution.

Traction Force Microscopy

To measure cell traction forces, PHH were seeded on stiff PAA gels embedded with 1 μl/mL red fluorescent beads (Fluorospheres, Invitrogen). Cells were cultured and treated with lipid as described. Brightfield images of cells on the gels and fluorescent images of the beads were taken and the locations marked on a Zeiss Axio Observer 7. Cells were dissociated with the addition of 1% Triton-X 100 for 10 min. Fluorescent images were taken again to determine the bead location without the cells attached. Bead images from before and after cell detachment were aligned using the Linear Stack Alignment with SIFT option of the Registration plugin in FIJI. Displacement fields of the beads were generated using the PIV plugin and then further analyzed with the FFTC plugin

to calculate forces. Cell outlines were traced from the brightfield images and used to define larger regions of interest (ROIs). Automatic thresholding was used to isolate the areas where traction forces were being generated in proximity to the cell boundary. These thresholded ROIs were used to measure mean and maximum traction forces.

Theoretical Modeling of Chromatin Condensation

To investigate the effect of epigenetic regulation and mechano-osmotic loading on the nucleus, we developed a mathematical model for chromatin phase-separation in the nucleus. The composition of the nucleus at any point and time was defined in terms of volume fractions heterochromatin $\phi_h(x,t)$, euchromatin $\phi_e(x,t)$ and nucleoplasm $\phi_n(x,t)$ such that $\phi_e+\phi_h+\phi_n=1$. Equivalently, the physical state of the nucleus at any point can be completely determined via two independent variables – ϕ_n, the volume fraction of nucleoplasm, and $\phi_d=\phi_h-\phi_e$, the difference between the volume fractions of heterochromatin and euchromatin. We constructed a free energy density function written as,

$$W(\phi_n,\phi_d,\nabla\phi_n,\nabla\phi_d)=\underbrace{\frac{k_BT}{\Omega}\phi_e^2+\frac{k_BT}{\Omega}(\phi_h-\bar{\phi}_h)^2\phi_h^2}_{\text{chromatin - chromatin interactions}}+\underbrace{\frac{\eta}{2}|\nabla\phi_w|^2+\frac{\eta}{2}|\nabla\phi_d|^2}_{\text{interfacial energy}}+\underbrace{V_l\phi_h e^{-d/d_o}}_{\text{chromatin-lamina interactions}} \quad (1)$$

The first two terms in Eq. (1) denote the energetic contributions arising from the competition between entropy and enthalpy of mixing of the two distinct phases – euchromatin phase with $\phi_h=0$ and heterochromatin phase with $\phi_h=\bar{\phi}_h$. The second term denotes the interfacial energies penalizing the formation of interfaces between the two phases, while the last term for $V_l<0$ captures the effect of proteins such as LAP2β, and LBR which mediate the interactions between chromatin and the nuclear lamina. Note that the chromatin-lamina interactions decrease with distance d from the lamina, over a length scale d_0.

The steady state chromatin organization in the nucleus was obtained as local minima of the total free energy defined using Eq. (1), giving rise to the governing equations of the steady state using variational principles as,

$$\begin{aligned}\mu_n(x,t)&=\frac{\partial W}{\partial\phi_n}-\nabla\cdot\left(\frac{\partial W}{\partial\nabla\phi_n}\right)\\ \mu_d(x,t)&=\frac{\partial W}{\partial\phi_d}-\nabla\cdot\left(\frac{\partial W}{\partial\nabla\phi_d}\right)\end{aligned} \quad (2)$$

where μ_n and μ_d are the chemical potentials of nucleoplasm and chromatin, respectively. Spatial gradients of the chemical potential of reactively inert nucleoplasm drive its spatio-temporal evolution via the diffusion kinetics as,

$$\frac{\partial\phi_n}{\partial t}=\underbrace{M_n\nabla^2\mu_n}_{\text{diffusion}} \quad (3)$$

where M_n is the mobility of nucleoplasm in the nucleus. The kinetics of chromatin evolution is driven by both the diffusion as well as epigenetic regulated reaction kinetics of acetylation and methylation as,

$$\frac{\partial\phi_d}{\partial t}=\underbrace{M_d\nabla^2\mu_d}_{\text{diffusion}}+\underbrace{2(\Gamma_{me}\phi_e-\Gamma_{ac}\phi_h)}_{\text{epigenetic regulation}} \quad (4)$$

where M_d is the mobility of chromatin in the nucleus and Γ_{me} and Γ_{ac} are the effective rates of methylation and acetylation of chromatin, respectively. Eq. (2), (3), and (4) together govern the organization of chromatin in the nucleus.

In addition, the nuclear envelope may enforce an interchange of water with the cytoplasm via water exchanging channels enforcing a chemical potential $\bar{\mu}_w$ at the nuclear periphery acting as the first boundary condition. Allowing water transport via chemical potential perturbation circumvents the need to know the location of individual nuclear pores and water channels in the nuclear lamina. Lastly, we assumed a no flux boundary condition along the nuclear periphery for chromatin kinetics. We introduced small perturbations to ϕ_d and ϕ_n around their initial values to initiate the separation of chromatin into the two phases, and let the simulation proceed until a steady state was reached.

Theoretical Modelling of Cytoskeletal/Lipid Droplet Interactions

To study the effect of lipid droplets on the chemo-mechanical behavior of cells, we used our theoretical cell model previously developed in reference (7). The three-dimensional cell model includes the following components: the cytoskeleton, the focal adhesions, and the nucleus (see reference (44) for details).

The cell cytoskeleton was treated as a continuum of representative volume elements (RVEs), each of which was comprised of (i) the myosin motors, (ii) the microtubules, and (iii) the actin filaments. Myosin molecular motors are the first element of the cytoskeleton in our model and generate internal contractility of the cell as experimentally reported(8). We treated the average density of phosphorylated myosin motors as a symmetric tensor ρ_{ij}, whose components represent cell contractility in different directions(9). The cell contractility ρ_{ij} generates compressive stress $C_{ijkl}^{(MT)}\varepsilon_{kl}^{(MT)}$ and tensile stress σ_{ij} in the cytoskeletal components that are in compression (e.g., microtubules) and tension (e.g., actin elements), respectively,

$$\rho_{ij} = -C_{ijkl}^{(X)}\varepsilon_{kl}^{(MT)} + \sigma_{ij} \qquad (5)$$

where $C_{ijkl}^{(MT)}$ and $\varepsilon_{kl}^{(MT)}$ are the stiffness and strain tensors of the cytoskeletal components that are in compression. Actin filaments are the second component of the cytoskeleton which are connected to the myosin element in series and subsequently experience tension and transmit myosin-generated tensile forces to the extracellular matrix through focal adhesions as experimentally observed(10-12). Microtubules are the third component of the cytoskeleton which are connected to the contractile myosin element in parallel and therefore experience compression consistent with experimental observations.

Focal adhesions in our coarse-grained model were modeled as a set of initially soft nonlinear mechanical elements that stiffen with tension to capture the tension-dependent formation of the focal adhesions. When the tensile stress exerted by the contractile cell to the adhesion layer exceeds a certain threshold, mature focal adhesions are formed, and the cell is connected to the substrate, while below this threshold, the stiffness of the adhesion layer remains low and the substrate experiences negligible forces. The nucleus was treated as an elastic thin layer (representing the nuclear envelope) filled with a solid elastic material representing chromatin and other subnuclear components. To perform traction force microscopy simulations, the cell model was coupled to the matrix model which treats the matrix substrate as a thick linear elastic material with an elastic modulus of 10 kPa as used in our traction force microscopy experiments. We treated the lipid droplets as growing mechanical inclusions within the cytoplasm. To this end, we modeled each droplet as a thin spherical membrane with internal pressure representing the enclosed fluid. We simulated the internal pressure by applying uniform and outward force spatially perpendicular to the internal surface of the membrane (outward arrows in Figure 5E). As a result of the internal pressure and droplet growth, the memebrane undergoes tensile stresses tangential to the membrane surface representing the surface tension in lipid droplets. In our model, lipid droplets can only have mechanical interactions with other cellular components and the model does not include any chemical effects of lipid droplets on cell behavior. We used a frictionless contact mode in our model for the contact between droplets and the nucleus as well as between droplets and the matrix substrate.

Statistics and Data Analysis

To avoid over confidence, statistics were performed on the biological replicates using a two-way analysis of variance (ANOVA) that fit a full-effect model followed by multiple comparisons with Tukey correction. In the case of the drug-treated cells in Figure 6 (where the n number for the drug-treated cells and controls were different), a two-way ANOVA that fit only a main-effects model was used, followed by a Fisher's test for multiple comparisons. Correlations were analyzed with linear regression followed by an F-test to determine whether the slope was significant. Analysis of covariance was run to compare the slope and intercept of the fit between groups. When slopes and intercepts were not significantly different, a pooled slope and intercept was calculated. The distribution of indent radii was compared between control and oleate treated cells using multiple

Kolmogorov–Smirnov (K-S) tests, one for each stiffness. The symmetry of indent distributions was determined by comparing the positive dent distribution with the absolute value of the negative dent distribution with a K-S test for each group. The frequency of γH2AX-positive nuclei was compared using multiple Chi-Squared tests comparing control to oleate-treated cells within a stiffness. Comparison of nuclear area and irregularity of fully γH2AX-positive nuclei was compared with an unpaired t-test. The prevalence of different numbers of actin fiber families was compared using multiple Chi-Squared tests comparing control to oleate-treated cells. Traction force measurements were compared using a two-way ANOVA with a full effect model on the biological replicates and the shown p-value is the column factor. Analysis of nuclear shape and HNF4α intensity in mouse liver tissue was done using a two-way ANOVA with a main effects model and the shown p-value is the column factor. For all graphs, significance values less than $p = 0.05$ are labeled. Color-coded significance bars indicate stiffness-dependent differences within a treatment group, either blue for BSA or green for oleate.

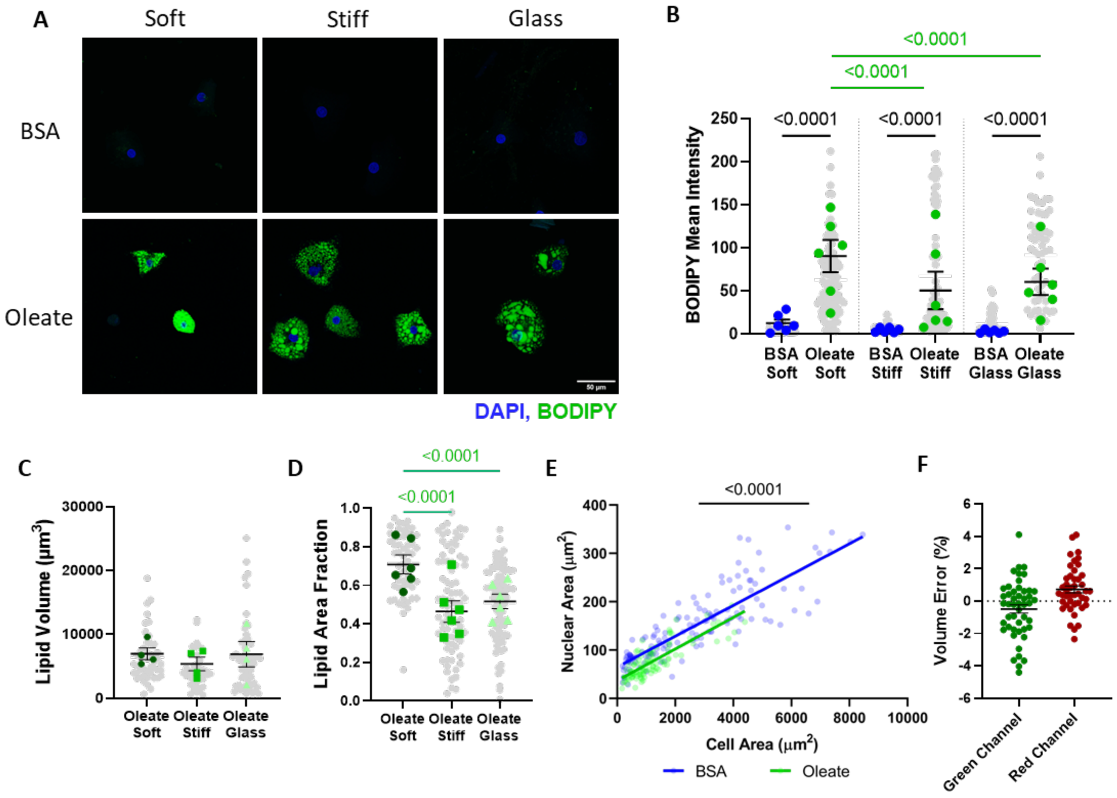

Fig. S1.

Oleate-treated cells accumulate significant amounts of lipid on all stiffness substrates. (**A**) Representative images of lipid accumulation in control and oleate-treated cells on soft PAA, stiff PAA, and glass. Nuclei stained with DAPI (Blue) and lipid with BODIPY (Green). Scale bar is 50um and is the same for all images. (**B**) BODIPY mean intensity for control and oleate-treated cells on soft PAA, stiff PAA, and glass as measured in maximum z-projections. (**C**) Lipid area fraction (lipid positive area/cell area) in oleate-treated cells on different stiffnesses as measured in maximum z-projections. (**D**) Lipid volume in oleate-treated cells on different stiffnesses as measured with 3D segmentation of z-stacks. (**E**) Scatter plot comparing cell area and nuclear area in individual cells, pooled across stiffnesses, fit with linear regression (n = 3). (**F**) Error in volume measurement of 15µm FocalCheck Beads as measured in both the green and red fluorescent channels. Statistics: (B, C) Cells are from n = 6 independent experiments. (D) Cells are from n = 4 independent experiments. P-values calculated with two-way ANOVA with multiple comparisons. (B-D) Colored dots are the means per experiment, while the grey dots are the individual cell values. (E) Data are the values for individual cells from $n = 3$ independent experiments. Blue indicates control cells, while green indicates oleate-treated cells. Slopes of both lines are significantly non-zero (P=<0.0001) but are not significantly different from one another. Y-intercepts are significantly different (P=<0.0001).

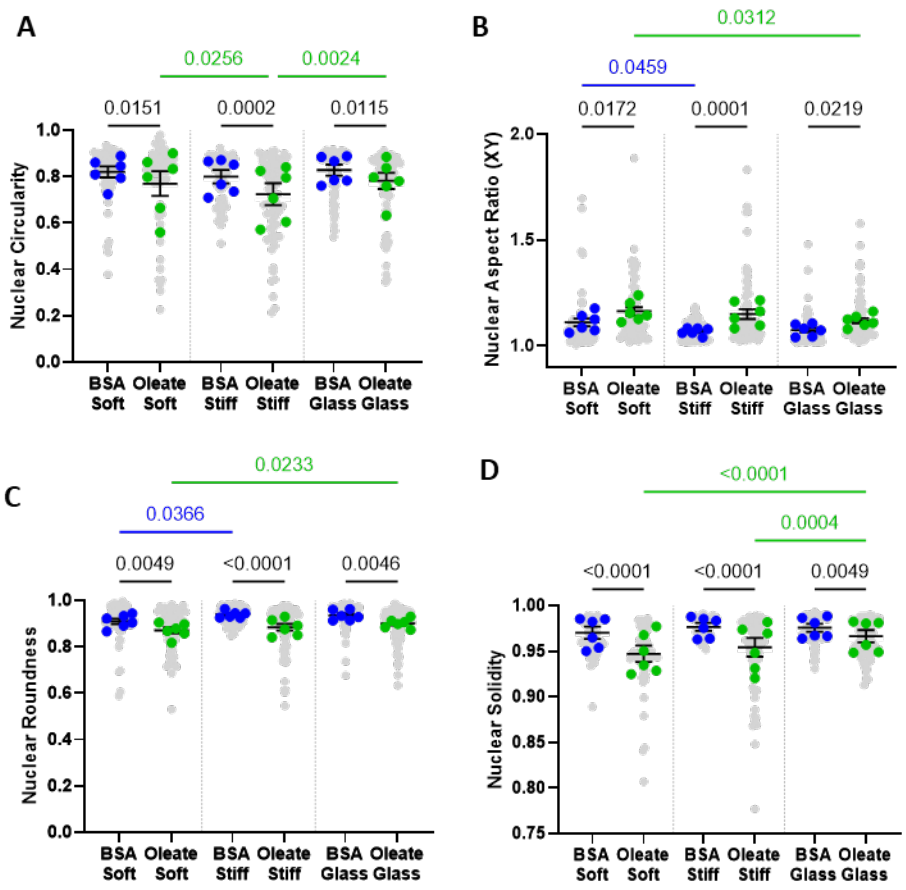

Fig. S2.

Nuclei of oleate-treated cells are highly deformed according to multiple shape parameters. (**A**) Nuclear circularity, (**B**) aspect ratio (in the XY plane), (**C**) roundness and (**D**) solidity in control and oleate-treated cells on different stiffness substrates. Statistics: Cells from *n* = *6* independent experiments, p-values calculated by two-way ANOVA with multiple comparisons.

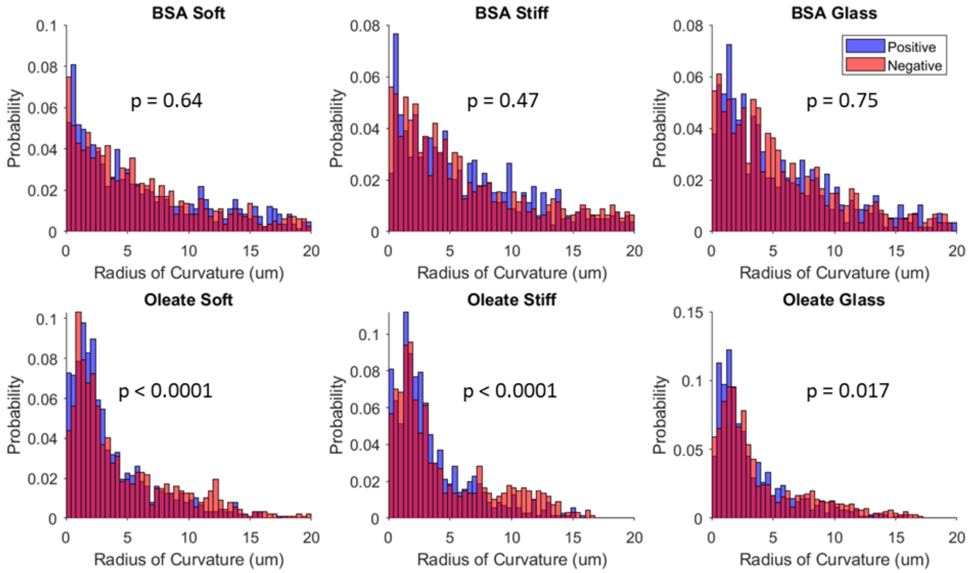

Fig. S3.
Distribution of radii of curvature for nuclear membrane indentations is symmetric for control but not oleate-treated cells. Overlaid histograms of the magnitude of positive (blue) and negative (orange) radii of curvature for control and oleate-treated cells on soft PAA, stiff PAA, and glass. Radii are all the indents from individual cells in $n = 3$ independent experiments. P-values calculated with K-S tests showing oleate-treated cells do not have symmetric distribution (distribution of positive and negative radii is not the same).

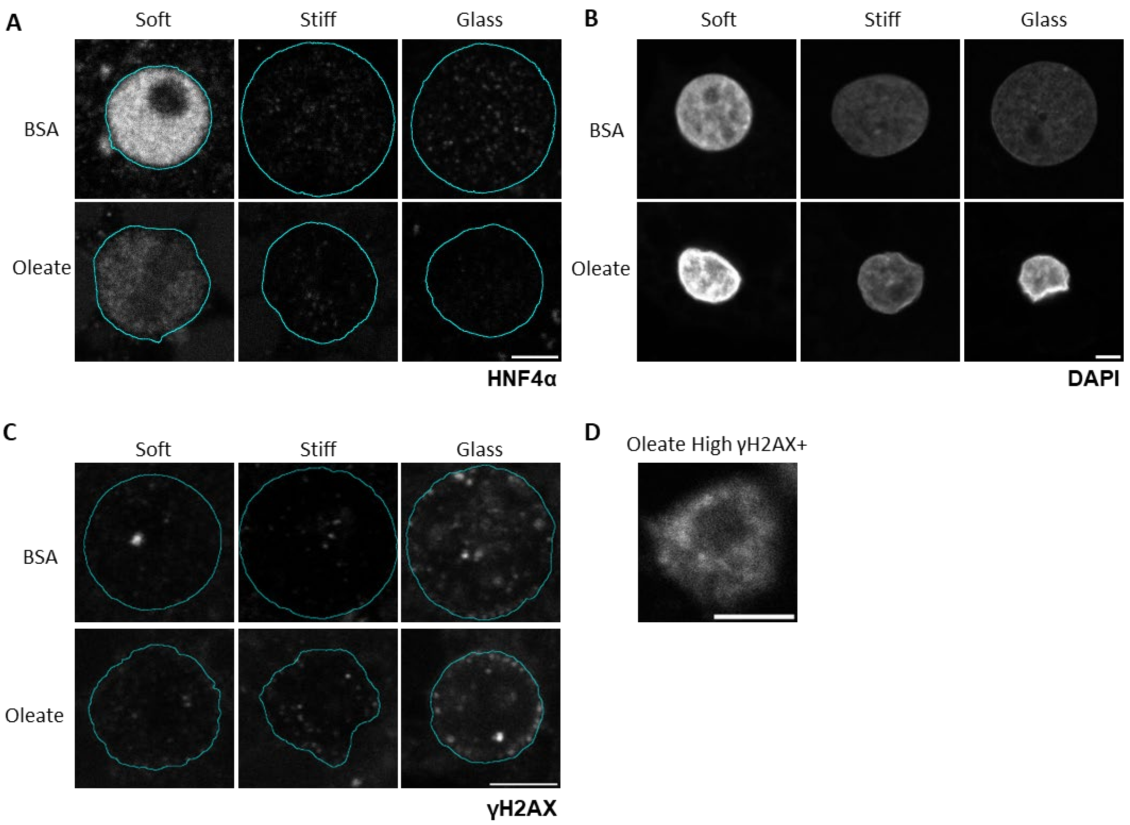

Fig. S4.
Lipid-loading reduces HNF4α and increases chromatin condensation, but does not increase DNA damage, except in extreme cases.
(**A**) Representative images of HNF4α intensity in control and oleate-treated cells on different stiffness substrates. Cyan outlines the nucleus, scale bar is 5μm and is the same for all images. (**B**) Representative images of DAPI stain used for analysis of chromatin condensation. Scale bar is 5μm and is the same for all images. (**C**) Representative images of γH2AX foci in control and oleate-treated cells on different stiffness substrates. Cyan outlines the nucleus, scale bar is 5μm and is the same for all images. (**D**) Representative image of high γH2AX signal in subset of oleate-treated cells, scale bar is 5μm.

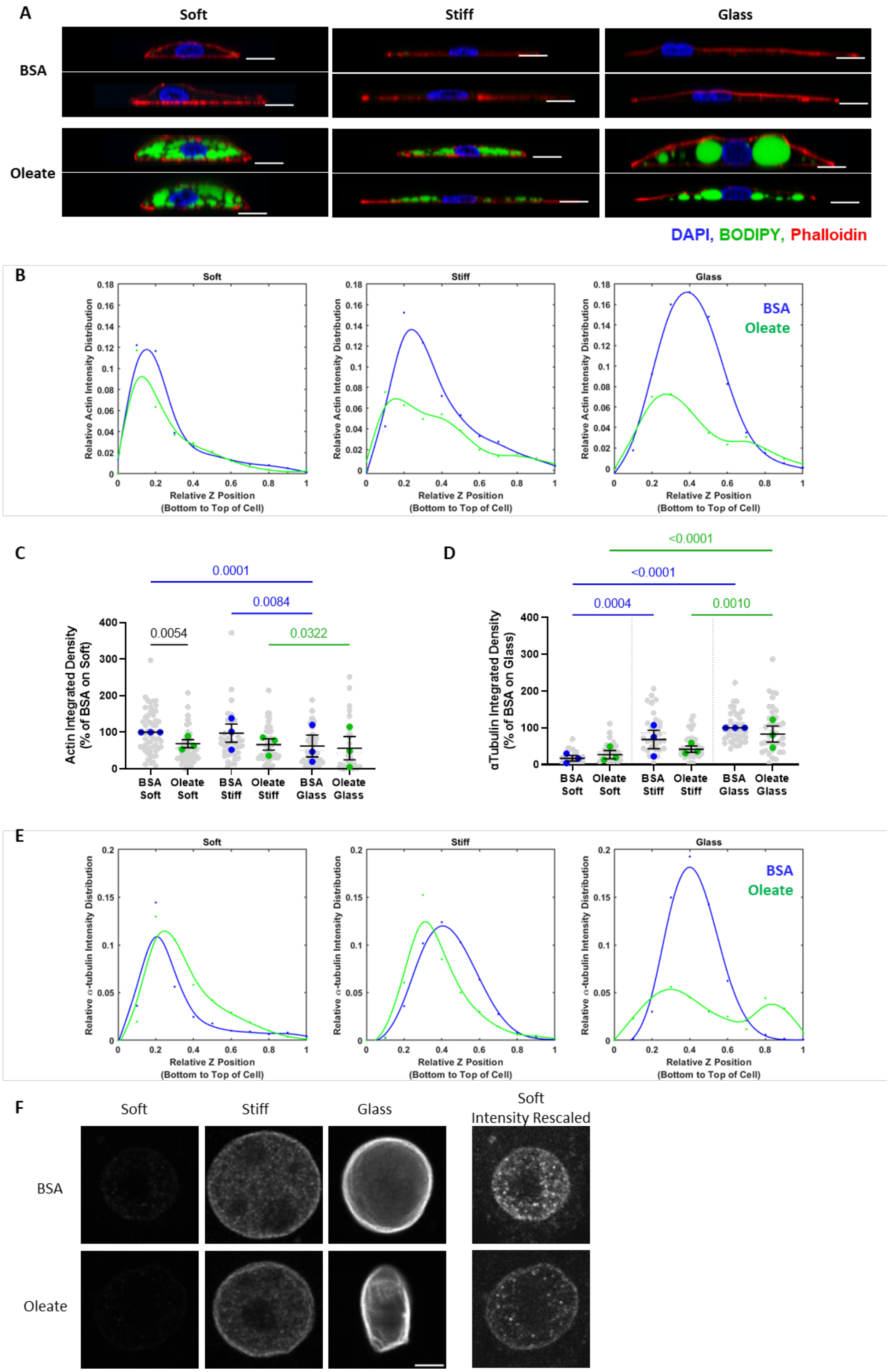
A
Soft
Stiff
Glass
BSA
Oleate
DAPI, BODIPY, Phalloidin
B
Relative Actin Intensity Distribution
Relative Z Position
(Bottom to Top of Cell)
BSA
Oleate
C
Actin Integrated Density
(% of BSA on Soft)
0.0001
0.0084
0.0054
0.0322
BSA Soft
Oleate Soft
BSA Stiff
Oleate Stiff
BSA Glass
Oleate Glass
D
αTubulin Integrated Density
(% of BSA on Glass)
<0.0001
<0.0001
0.0004
0.0010
E
Relative α-tubulin Intensity Distribution
Relative Z Position
(Bottom to Top of Cell)
F
Soft
Stiff
Glass
Soft
Intensity Rescaled
BSA
Oleate

Fig. S5.

Lipid droplets alter the apical-basal distribution of cytoskeletal fibers. (**A**) Representative YZ cross-sectional images of actin distribution in control and oleate- treated cells on different stiffness substrates. DAPI (Blue), BODIPY (Green), and Phalloidin (Red). Scale bar is 10µm. (**B**) Distribution of phalloidin integrated density from the basal to apical membrane of control and oleate-treated cells on soft PAA, stiff PAA, and glass, fit with a smoothing-spline. (**C**) Phalloidin integrated density, indicating total actin content, in control and oleate-treated cells. (**D**) α-tubulin integrated density, indicating total microtubule content, in control and oleate-treated cells. (**E**) Distribution of alpha-tubulin integrated density from the basal to apical membrane of control and oleate-treated cells on soft PAA, stiff PAA, and glass, fit with a smoothing-spline. (**F**) Representative images of lamin A/C staining in in control and oleate- treated cells on different stiffness substrates. Scale bar is 5µm and is the same for all images. To compare between treatments on soft, the images in the far right column are the same as the images in the far left column, just with the intensity rescaled. Intensity was rescaled to the same value for both images. Statistics: (B-D) Cells for all panels from *n = 3* independent experiments, p-values calculated by two-way ANOVA with multiple comparisons.

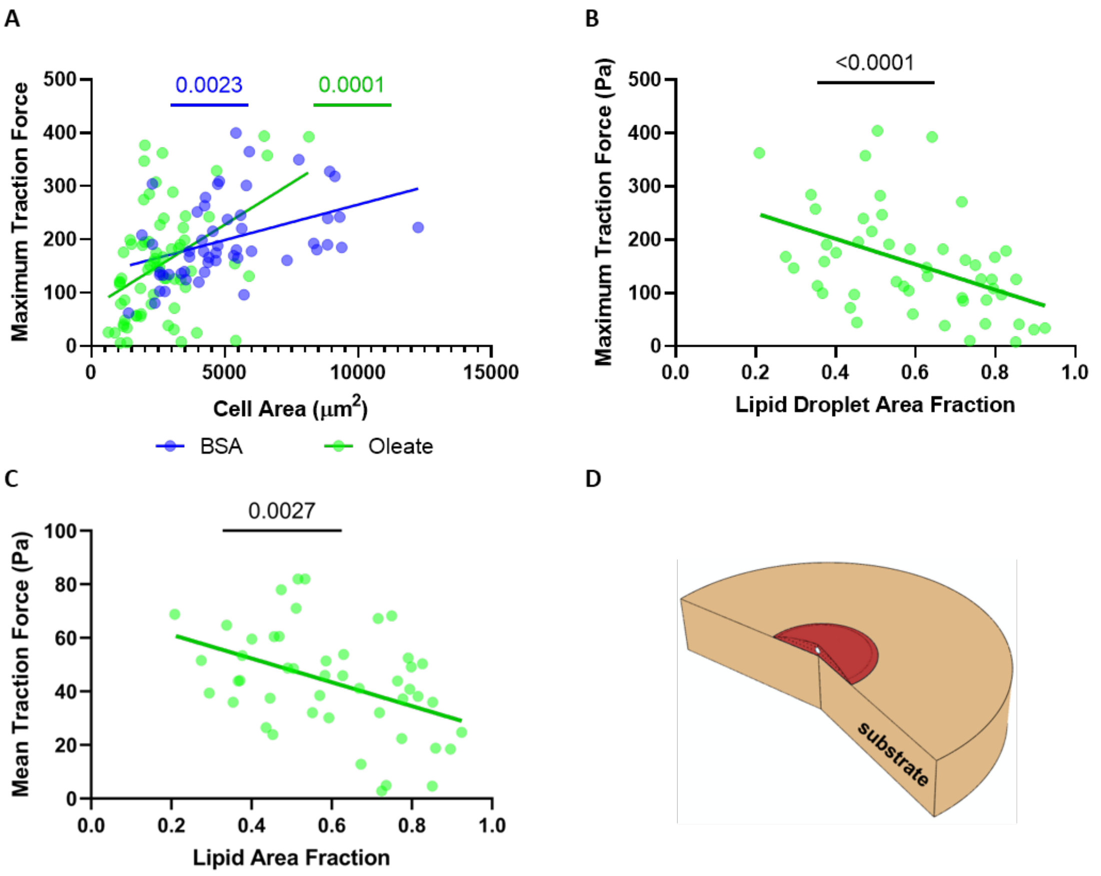

Fig. S6.

Maximum traction forces decrease proportional to lipid-loading. (**A**) Scatter plot with linear regression of cell area versus maximum traction force in individual cells for control and oleate-treated cells. (**B**) Scatter plot and linear regression of lipid droplet density and maximum traction force in oleate-treated cells. (**C**) Scatter plot and linear regression of lipid droplet density and mean traction force. (**D**) Geometry of the cell and substrate over which cell traction forces were modelled, red indicating the cell and tan indicating the substrate. Data are individual cells from $n = 3$ independent experiments. P-values calculated with F-test.

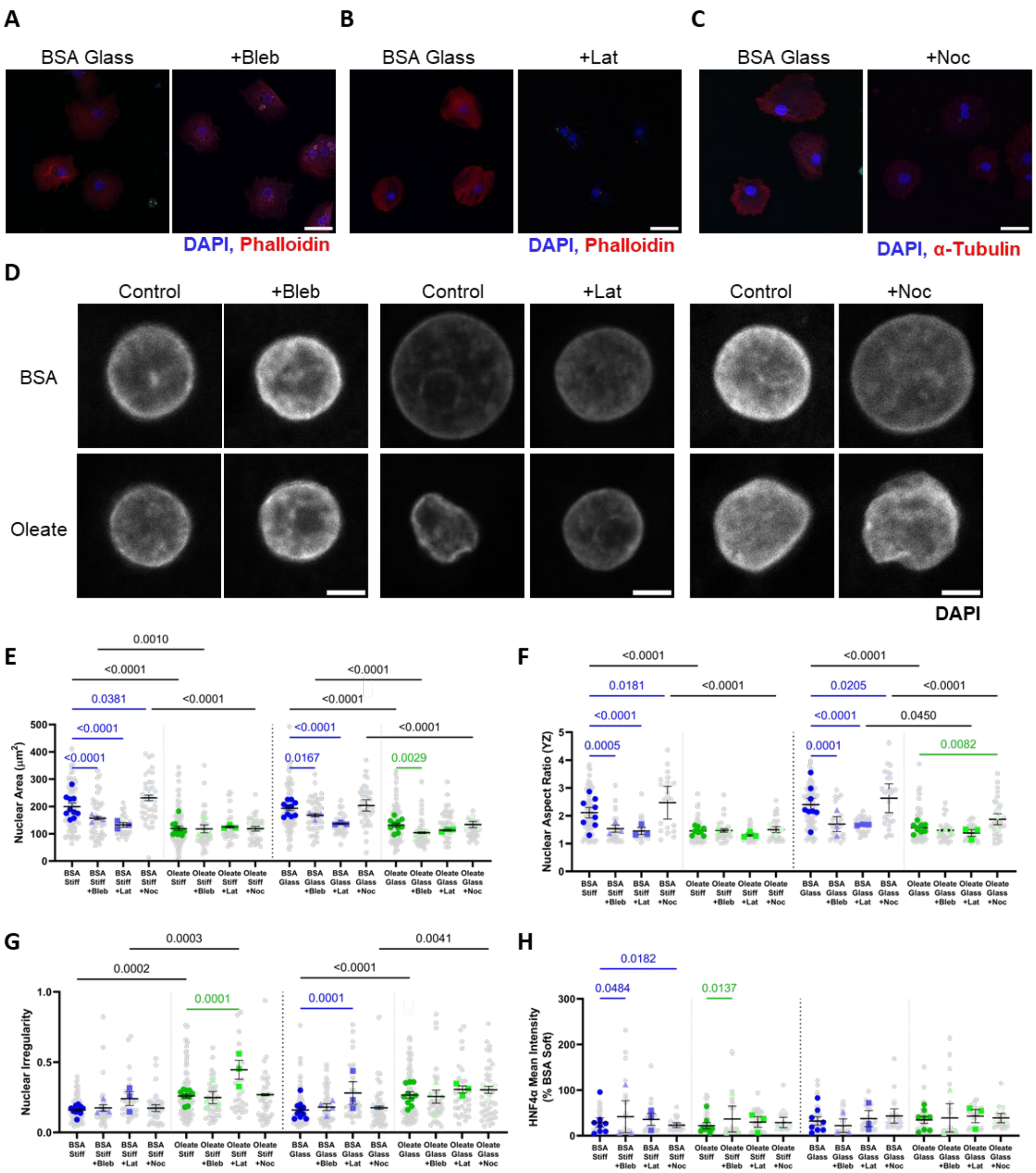

Fig. S7.

Lipid-loaded nuclei exhibit fewer morphological changes in response to cytoskeletal perturbation. Representative images showing effective cytoskeletal perturbation with (**A**) blebbistatin, (**B**) latrunculin, and (**C**) nocodazole. Scale bar is 50um and is the same for all images in each panel. (**D**) Representative DAPI images in control and oleate-treated cells on soft substrates treated with various cytoskeletal drugs. Scale bar is 5µm and is the same for all images. Blebbistatin (Bleb), Latrunculin A (Lat), Nocodazole (Noc). (**E**) Nuclear area, (**F**) cross-sectional nuclear aspect ratio, (**G**) nuclear irregularity, and (**H**) mean HNF4α intensity (normalized to control on soft) in control and oleate-treated cells with or without the addition of cytoskeletal drugs on stiff PAA and glass. Statistics: (G-H) Data are the mean +- s.e. of $n = 3$ independent experiments for each drug treatment and $n = 8$ for the non-treated. P-values were calculated using two-way ANOVA with a main effects model with multiple comparisons. Shades of blue indicate control cells, while shades of green indicate oleate-treated cells.

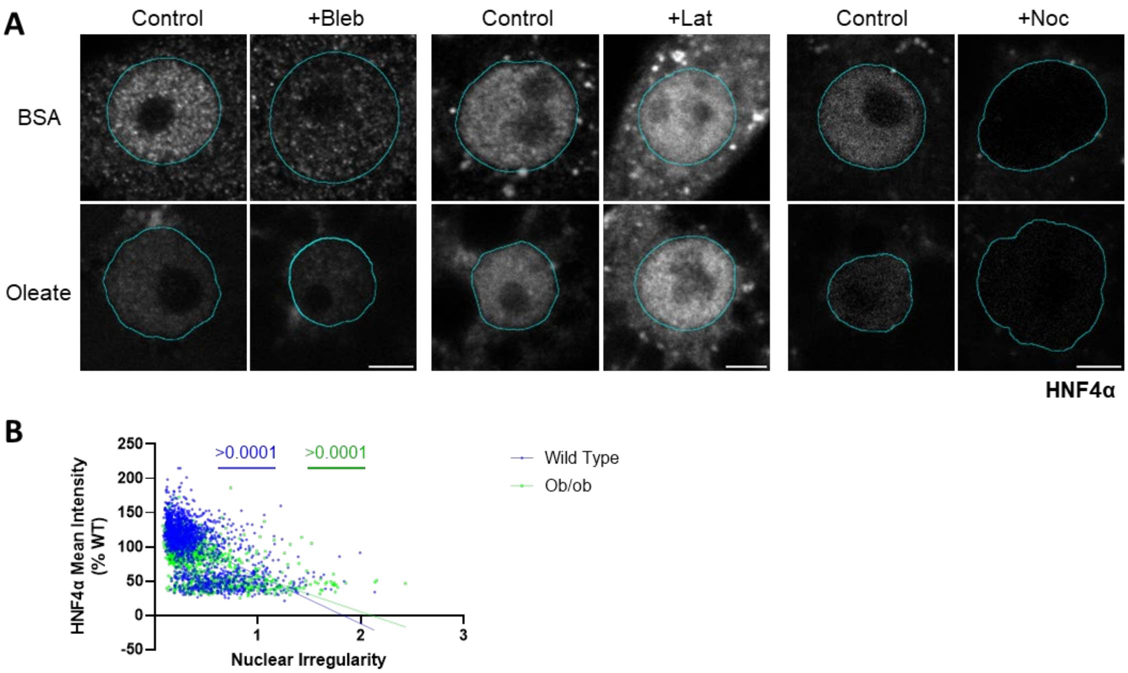

Fig S8.
HNF4α is altered by cytoskeletal perturbation in culture and reduced in mouse model of steatosis.
(**A**) Representative images of HNF4α intensity in control and oleate-treated cells on soft substrates treated with various cytoskeletal drugs. Cyan outlines the nucleus segmented in the DAPI channel. Scale bar is 5μm and is the same for all images. Blebbistatin (Bleb), Latrunculin A (Lat), Nocodazole (Noc). (**B**) Scatter plot with linear regression of nuclear irregularity vs. mean HNF4α intensity of all cells from wild type and ob/ob mouse livers. Statistics: P-values calculated with an F-test.

Movie S1 (separate file). Z-stack of microtubule organization in control primary human hepatocyte on glass. **Nuclei stained with DAPI (Blue), microtubules with α-tubulin (Red) and lipid with BODIPY (Green). Scale bar is 15μm.**

Movie S2 (separate file). Z-stack of microtubule organization in oleate-treated primary human hepatocyte on glass. **Nuclei stained with DAPI (Blue), microtubules with α-tubulin (Red) and lipid with BODIPY (Green). Scale bar is 15μm.**

Supplemental References

1. M. Guo *et al.*, Cell volume change through water efflux impacts cell stiffness and stem cell fate. *Proc Natl Acad Sci U S A* **114**, E8618-E8627 (2017).
2. C. Roffay *et al.*, Passive coupling of membrane tension and cell volume during active response of cells to osmosis. *Proc Natl Acad Sci U S A* **118** (2021).
3. J. Irianto, D. A. Lee, M. M. Knight, Quantification of chromatin condensation level by image processing. *Med Eng Phys* **36**, 412-417 (2014).
4. J. Irianto *et al.*, Osmotic challenge drives rapid and reversible chromatin condensation in chondrocytes. *Biophys J* **104**, 759-769 (2013).

5. M. Witte, S. Jaspers, H. Wenck, M. Rubhausen, F. Fischer, General method for classification of fiber families in fiber-reinforced materials: application to in-vivo human skin images. *Sci Rep* **10**, 10888 (2020).
6. N. Chernov (2009) Circle Fit (Pratt Method). (MATLAB Central File Exchange).
7. F. Alisafaei, D. S. Jokhun, G. V. Shivashankar, V. B. Shenoy, Regulation of nuclear architecture, mechanics, and nucleocytoplasmic shuttling of epigenetic factors by cell geometric constraints. *Proc Natl Acad Sci U S A* **116**, 13200-13209 (2019).
8. M. S. Kolodney, E. L. Elson, Correlation of myosin light chain phosphorylation with isometric contraction of fibroblasts. *J Biol Chem* **268**, 23850-23855 (1993).
9. V. B. Shenoy, H. Wang, X. Wang, A chemo-mechanical free-energy-based approach to model durotaxis and extracellular stiffness-dependent contraction and polarization of cells. *Interface Focus* **6**, 20150067 (2016).
10. S. Kumar *et al.*, Viscoelastic retraction of single living stress fibers and its impact on cell shape, cytoskeletal organization, and extracellular matrix mechanics. *Biophys J* **90**, 3762-3773 (2006).
11. K. Katoh, Y. Kano, M. Masuda, H. Onishi, K. Fujiwara, Isolation and contraction of the stress fiber. *Mol Biol Cell* **9**, 1919-1938 (1998).
12. M. Ghibaudo *et al.*, Traction forces and rigidity sensing regulate cell functions. *Soft Matter* **4**, 1836-1843 (2008).
